# Towards the total synthesis of keramaphidin B

**DOI:** 10.3762/bjoc.12.104

**Published:** 2016-05-30

**Authors:** Pavol Jakubec, Alistair J M Farley, Darren J Dixon

**Affiliations:** 1 Department of Chemistry, Chemistry Research Laboratory, University of Oxford, 12 Mansfield Road, Oxford, OX1 3TA, United Kingdom

**Keywords:** bifunctional organocatalyst, enantioselective Michael addition, keramaphidin B, nitro-Mannich lactamisation cascade

## Abstract

The enantio- and diastereoselective Michael addition of a δ-valerolactone-derived pronucleophile to a substituted furanyl nitroolefin catalysed by a bifunctional cinchonine-derived thiourea has been used as the key stereocontrolling step in a new synthetic strategy to the heavily functionalised piperidine core of keramaphidin B.

## Introduction

**Figure 1:**
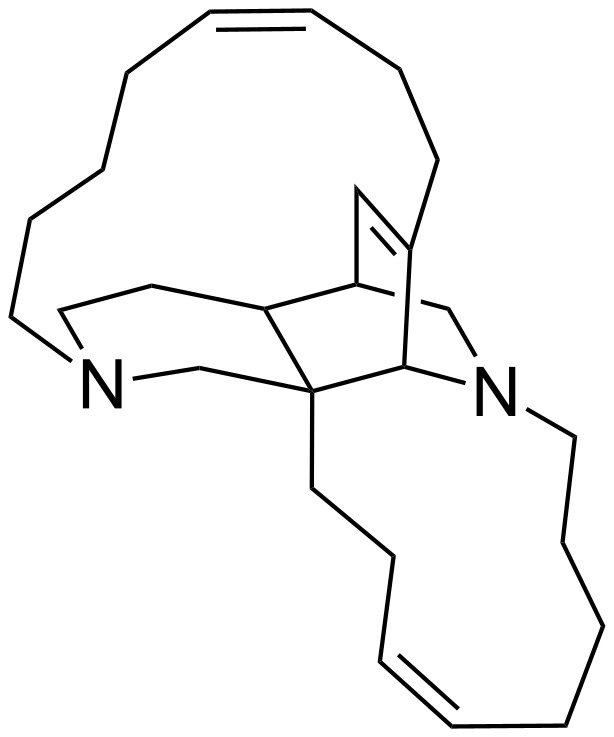
Keramaphidin B (**1**).

Keramaphidin B (**1**) is a marine alkaloid first isolated by Kobayashi in 1994 from the Okinawan marine sponge *Amphimedon* sp and has been shown to be cytotoxic against KB human epidermoid carcinoma cells (IC_50_ 0.28 μg/mL) and P388 murine leukemia cells (IC_50_ 0.28 μg/mL) [1]. It is a member of the manzamine alkaloids and has an exquisite molecular structure comprising a 6,6,6,11,13 pentacycle possessing 4 stereogenic centres including one quaternary centre (Figure 1). In 1992, two years before its isolation, Baldwin and Whitehead, in their landmark paper entitled ‘On the Biosynthesis of Manzamines’ postulated that keramaphidin B was a common intermediate in the biosynthesis of the manzamine alkaloids [2]. Several years later, Baldwin and co-workers synthesised keramaphidin B following a biomimetic pathway via an intramolecular Diels–Alder reaction as the late stage key step. After extensive purification, the authors were able to isolate keramaphidin B in just 0.3% yield, but nevertheless they provided evidence for the biosynthesis [3]. A year later, Baldwin et al. completed an alternative synthesis by performing an intermolecular Diels–Alder reaction and a double late stage RCM reaction to close the two macrocyclic rings; albeit the last stage afforded **1** in 1% yield after separation of various oligomeric byproducts [4].

Our group has had a long-standing research program dedicated towards the total syntheses of the manzamine alkaloids with a particular emphasis on the development of novel catalytic methods for simplifying their syntheses [5–10]. Owing to keramaphidin B’s attractive structure combined with its interesting biological profile and the lack of an efficient method for its synthesis, we selected **1** as a suitable synthetic target. Our plan was to design and implement a new synthetic route that integrated some of our newly developed bifunctional organocatalytic reactions, as well as cascade technologies, to rapidly and stereoselectively build up the core of this fascinating molecule. Herein we wish to report our preliminary synthetic efforts towards the stereoselective synthesis of the heavily functionalised piperidine core of keramaphidin B (**1**).

## Results and Discussion

**Figure 2:**
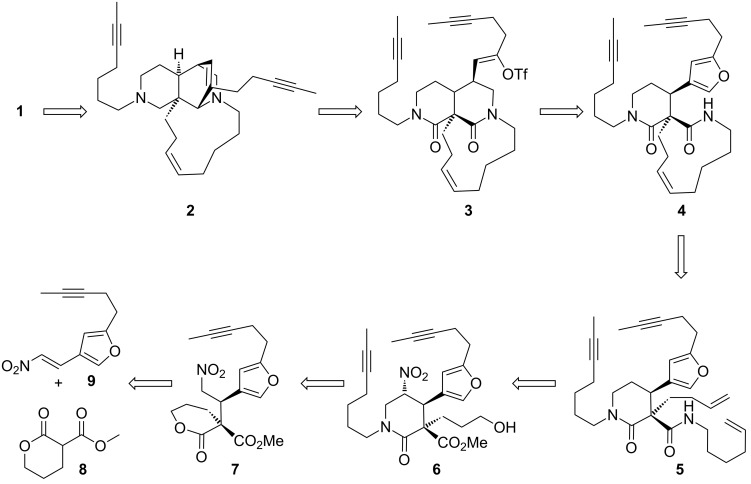
Retrosynthetic analysis of keramaphidin B.

Our overall synthetic strategy is presented in Figure 2. We envisaged that a late stage alkyne RCM reaction of **2** and *cis*-selective hydrogenation of the internal alkyne would present an efficient method for the synthesis of the 13-membered ring.

The synthesis of the 11-membered ring could be achieved by a *Z*-selective alkene RCM reaction [5] to afford spirocyclic bislactam **4** from metathesis precursor **5**. Bisalkene **5** could in turn be synthesised by an aminolysis/oxidation/olefination sequence of the terminal alcohol **6**, following traceless nitro group removal.

5-Nitropiperidin-2-one **6** in turn could be accessed by a nitro-Mannich lactamisation cascade reaction between Michael adduct **7**, formaldehyde and a suitable primary amine, following our well-established protocol [6–12]. The key quaternary stereocentre of keramaphidin B, we envisaged, would be installed through an enantio- and diastereoselective organocatalytic Michael addition [13–15] between pronucleophile **8** and the known substituted furanyl nitroolefin **9** under the control of a cinchona-derived bifunctional Brønsted base/H-bond donor organocatalyst developed in our group and others [16–19].

### Bifunctional organocatalysed Michael addition studies

**Scheme 1:**
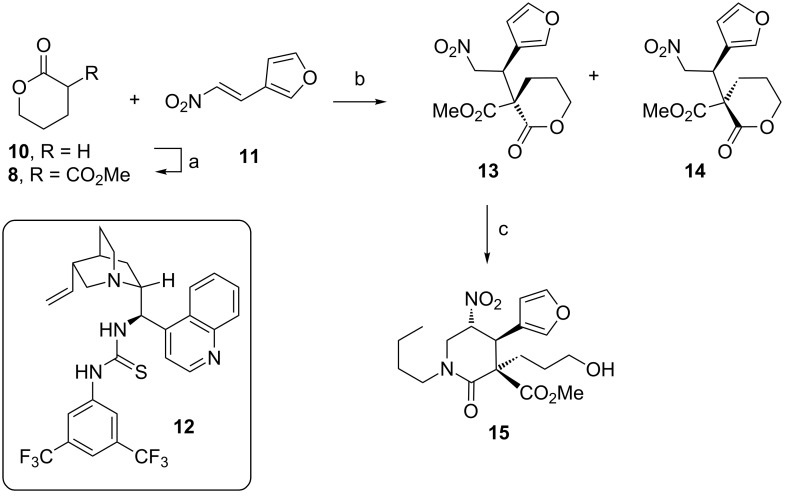
Enantio- and diastereoselective bifunctional thiourea **12** organocatalysed Michael addition. (a) CO(OMe)_2_, LHMDS, THF, −78 °C to rt, 83%; (b) 20 mol % **12**, toluene, −20 °C, 24 h, 95:5 dr (**13**:**14**), 90:10 er for **13**, 99% yield; (c) butylamine, aq formaldehyde, MeOH, reflux, 1 h, 63%.

In our previous total syntheses of nakadomarin A [5,7,20] and manzamine A [10] the stereochemical configuration of the quaternary carbon was established by a diastereoselective Michael addition between a chiral, single enantiomer, cyclic β-amido ester and a nitroolefin, and, in the case of nakadomarin A the reaction could be rendered catalytic using a bifunctional cinchonine-derived urea catalyst. We reasoned that a similar catalytic approach could be used to fix the absolute stereochemical configuration in the Michael addition reaction between δ-valerolactone pronucleophile **8** and nitro-olefin **9**, although a degree of uncertainty as to the relative stereochemical outcome of the catalyst-controlled Michael reaction remained present. Accordingly, we chose to probe reactivity and establish relative stereocontrol using a close model system comprising pronucleophile **8** and furanyl nitroolefin **11** (Scheme 1).

The δ-valerolactone pronucleophile **8** was synthesised by the enolate acylation of δ-valerolactone (**10**) with dimethyl carbonate, using LHMDS as the base, in 83% yield. The furanyl nitroolefin **11** was readily synthesised on multigram scale via a Henry condensation, according to literature procedures [20]. We were delighted to observe that the Michael addition reaction using our previously reported cinchonine-derived bifunctional thiourea catalyst **12** afforded the addition product **13** in high yield with good levels of diastereo- and enantioselectivity (95:5 dr, 90:10 er for the major diastereomer **13**). The relative stereochemical configuration of the minor diastereomeric product **14** was determined by single X-ray crystallographic analysis of *rac-***14** (see Supporting Information File 1) and revealed that the major diastereomer **13** indeed possessed the necessary stereochemical configuration [21] for accessing keramaphidin B, assuming the chemoselectivity of the nitro-Mannich lactamisation favoured attack at the more reactive δ-lactone carbonyl instead of that of the methyl ester.

Pleasingly, this was indeed realised at the next stage; performing a nitro-Mannich lactamisation cascade on **13** with formaldehyde and butylamine in methanol afforded lactam **15** in 63% yield, possessing the hydroxypropyl chain attached to the quaternary stereocentre, poised for further functionalisation.

**Scheme 2:**
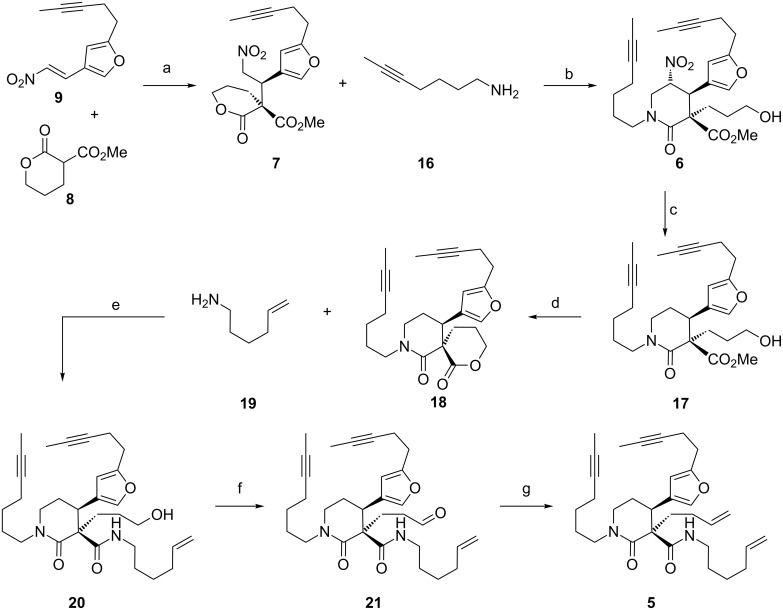
Synthesis of bis alkene **5**. (a) **12** (20 mol %), toluene, −20 °C, 36 h, 95:5 dr, 92% yield; (b) aq HCHO, MeOH, reflux, 70 °C, 1 h, >95:5 dr, 91:9 er, 56% yield; (c) AIBN, Bu_3_SnH, toluene, reflux, 0.33 h, 71% yield; (d) Ti(OiPr)_4_, toluene, 50 °C, 2 h, 84% yield; (e) **19** (neat), 130 °C, 24 h, 67% yield; (f) (COCl)_2_, DMSO, NEt_3_, CH_2_Cl_2_, −78 °C to rt, 0.5 h, 88% yield; (g) Cp_2_Ti(CH_3_)_2_, THF/toluene, reflux, 0.33 h, 42% yield.

Having established that the cinchonine-derived bifunctional Brønsted base/thiourea organocatalyst **12** was effective for installing two stereocentres including the quaternary carbon in a model system, we next performed the reaction using the substituted furanyl nitroolefin **9** (Scheme 2).

The organocatalysed Michael addition of **8** with substituted furanyl nitroolefin **9** [20] under the control of bifunctional organocatalyst **12** proceeded efficiently and, although the reaction time was slightly increased relative to the model system, identical levels of diastereo- and enantiocontrol were observed in the formation of **7** (92% yield). Treatment of the major diastereomeric product **7** with hept-5-yn-1-amine (**16**) and formaldehyde in boiling methanol afforded the lactam **6** in 56% yield as a single diastereomer in 91:9 er. Having played a key role in the Michael addition reaction and the nitro-Mannich lactamisation cascade, at this stage the nitro functionality had fully served its purpose. Accordingly, traceless reductive cleavage of the nitro group [22] using tributyltin hydride and AIBN was carried out to afford piperidin-2-one **17** in 71% yield. Lactonisation under Lewis acidic conditions afforded spirocyclic malonamate **18** possessing the correct relative stereochemistry for keramaphidin B, in 84% yield. Aminolysis under neat conditions with hex-5-en-1-amine (**19**) gave the primary alcohol **20** (67% yield), which was subsequently subjected to a Swern oxidation to yield the aldehyde **21** in 88% yield. Finally, treatment of the aldehyde with Petasis reagent afforded the target bisalkene RCM precursor **5** in a satisfactory 42% yield.

## Conclusion

In conclusion we have utilized a bifunctional cinchona*-*derived thiourea organocatalyst **12** for governing the key Michael addition towards the synthesis of keramaphidin B. The catalyst imparted high levels of enantio- and diastereocontrol at the newly formed contiguous tertiary and quaternary stereocentres. The stereochemical integrity of the newly formed stereogenic centres was not compromised during a subsequent three-step nitro-Mannich lactamisation cascade, aminolysis and lactonisation sequence. Further manipulation of the pendant functional groups allowed the synthesis of target compound **5** bearing two alkynes and two terminal alkenes for successive RCM reactions to construct the 11- and 13-membered rings of keramaphidin B. RCM precursor **5** – possessing all of the necessary masked functionality already installed about a piperidin-2-one framework – represents an advanced intermediate for the potential future synthesis of keramaphidin B, and our work towards this goal will be reported in due course.

## Supporting Information

File 1 Experimental procedures, analytical data, copies of NMR spectra and single X-ray crystal diffraction data of **14**.

## References

[1] Kobayashi J, Tsuda M, Kawasaki N, Matsumoto K, Adachi T (1994). Tetrahedron Lett.

[2] Baldwin J E, Whitehead R C (1992). Tetrahedron Lett.

[3] Baldwin J E, Claridge T D W, Culshaw A J, Heupel F A, Lee V, Spring D R, Whitehead R C, Boughtflower R J, Mutton I M, Upton R J (1998). Angew Chem, Int Ed.

[4] Baldwin J E, Claridge T D W, Culshaw A J, Heupel F A, Lee V, Spring D R, Whitehead R C (1999). Chem – Eur J.

[5] Yu M, Wang C, Kyle A F, Jakubec P, Dixon D J, Schrock R R, Hoveyda A H (2011). Nature.

[6] Jakubec P, Helliwell M, Dixon D J (2008). Org Lett.

[7] Jakubec P, Cockfield D M, Dixon D J (2009). J Am Chem Soc.

[8] Pelletier S M-C, Ray P C, Dixon D J (2009). Org Lett.

[9] Pelletier S M-C, Ray P C, Dixon D J (2011). Org Lett.

[10] Jakubec P, Hawkins A, Felzmann W, Dixon D J (2012). J Am Chem Soc.

[11] Jakubec P, Cockfield D M, Helliwell M, Raftery J, Dixon D J (2012). Beilstein J Org Chem.

[12] Clark P G K, Vieira L C C, Tallant C, Fedorov O, Singleton D C, Rogers C M, Monteiro O P, Bennett J M, Baronio R, Müller S (2015). Angew Chem, Int Ed.

[13] Marqués-López E, Herrera R P, Christmann M (2010). Nat Prod Rep.

[14] Tsogoeva S B (2007). Eur J Org Chem.

[15] Chen D Y-K, Ma D (2013). Beilstein J Org Chem.

[16] Ye J, Dixon D J, Hynes P S (2005). Chem Commun.

[17] Li B-J, Jiang L, Liu M, Chen Y-C, Ding L-S, Wu Y (2005). Synlett.

[18] McCooey S H, Connon S J (2005). Angew Chem, Int Ed.

[19] Vakulya B, Varga S, Csámpai A, Soós T (2005). Org Lett.

[20] Kyle A F, Jakubec P, Cockfield D M, Cleator E, Skidmore J, Dixon D J (2011). Chem Commun.

[R21] 21The absolute stereochemical configuration of the tertiary stereocentre was assigned by analogy; see references [7,11,16,20].

[22] Ono N, Kaji A (1986). Synthesis.

