# Supplementary material for: Towards the total synthesis of keramaphidin B
Source: Beilstein J Org Chem. 2016 May 30;12:1096–100. doi: 10.3762/bjoc.12.104 (PMC4901993; doi:10.3762/bjoc.12.104)
Supplement: File 1 — Experimental procedures, analytical data, copies of NMR spectra and single X-ray crystal diffraction data of 14. [file Beilstein_J_Org_Chem-12-1096-s001.pdf]

Supporting Information

for

Towards the total synthesis of keramaphidin B

Pavol Jakubec[1], Alistair J. M. Farley[1] and Darren J. Dixon*[1]

Address: [1]Department of Chemistry, Chemistry Research Laboratory, University of Oxford, 12 Mansfield Road, Oxford, OX1 3TA, United Kingdom

Email: Darren J. Dixon - darren.dixon@chem.ox.ac.uk

* Corresponding author

Experimental procedures, analytical data, copies of NMR spectra and single X-ray crystal diffraction data of 14

1 General experimental S3

1.1 Solvents and reagents S3

1.2 Chromatography S3

1.3 Instrumentation S3

1.4 Starting materials S4

2 Practical experimental S5

2.1 Synthesis and characterization of **8** S5

2.2 Synthesis and characterization of **13** and **14** S6

2.3 Synthesis and characterization of *rac*-**13** and *rac*-**14** S7

2.4 Synthesis and characterisation of **15** S8

2.5 Synthesis and characterisation of **7** and **7’** S9

2.6 Synthesis and characterisation of **6** S10

2.7 Synthesis of *rac*-**6** S11

2.8 Synthesis and characterization of **17** S12

2.9 Synthesis and characterisation of **18** S13

2.10 Synthesis and characterisation of **20** S14

2.11 Synthesis and characterisation of **21** S15
2.12 Synthesis and characterisation of **5** S16
3 Single X-ray Crystal Diffraction Data of 14 .... S17
4 References S22
5 Supplementary Data S23
5.1 ^{1}H and ^{13}C NMR spectra of **8** S23
5.2 ^{1}H and ^{13}C NMR spectra of **13** S24
5.3 ^{1}H and ^{13}C NMR spectra of **14** S25
5.4 ^{1}H and ^{13}C NMR spectra of **15** S26
5.5 ^{1}H and ^{13}C NMR spectra of **7** S27
5.6 ^{1}H and ^{13}C NMR spectra of **6** S28
5.7 ^{1}H and ^{13}C NMR spectra of **17** S29
5.8 ^{1}H and ^{13}C NMR spectra of **18** S30
5.9 ^{1}H and ^{13}C NMR spectra of **20** S31
5.10 ^{1}H and ^{13}C NMR spectra of **21** S32
5.11 ^{1}H and ^{13}C NMR spectra of **5** S33
5.12 HPLC chromatogram of **13** S34
5.13 HPLC chromatogram of **6** S35

1 General experimental

1.1 Solvents and reagents

Concentration under reduced pressure was performed by rotary evaporation at the appropriate pressure and temperature. Reagents used were obtained from commercial suppliers or purified according to standard procedures. Petroleum ether refers to distilled light petroleum of fraction 30–40 °C. Anhydrous toluene, tetrahydrofuran, dichloromethane and diethyl ether were dried by filtration through activated alumina (powder ~150 mesh, pore size 58 Å, basic, Sigma-Aldrich) columns. Dimethyl sulfoxide and dimethylformamide were used as supplied. Deuterated solvents were used as supplied.

1.2 Chromatography

Reactions were monitored by thin-layer chromatography (TLC) using Merck silica gel 60 F_{254} plates and visualized by fluorescence quenching under UV light. In addition, TLC plates were stained with potassium permanganate solution. Flash column chromatography (FCC) was performed on VWR 60 silica gel 40–63 μm using technical grade solvents that were used as supplied.

1.3 Instrumentation

Melting points were obtained on a Leica Galen III Hot-stage melting point apparatus and microscope and on a Kofler hot block and are reported uncorrected. All ^{1}H and ^{13}C NMR spectra were recorded using a Bruker 500 MHz and Bruker 400 MHz spectrometers and are quoted in ppm for measurement against a residual solvent peaks as internal standards. Chemical shifts (δ) are given in parts per million (ppm) and coupling constants (*J*) are given in Hertz (Hz). The ^{1}H NMR spectra are reported as follows: δ [multiplicity (s = singlet, d = doublet, t = triplet, q = quartet, quin = quintet, m = multiplet, "t" = apparent triplet, "d" = apparent doublet, "q" = apparent quartet, br. s = broad singlet, br. d = broad doublet, sxt = sextet), number of protons, coupling constants *J*/Hz (where appropriate) and assignment (where appropriate)]. DEPT 135 and two-dimensional (COSY, HSQC, HMBC) NMR spectroscopy were used where appropriate to assist the assignment of signals in the ^{1}H and ^{13}C NMR spectra. Low resolution mass spectra were recorded on a Waters LCT premier XE Micromass spectrometer (ESI). High-resolution mass spectra were recorded on a Bruker

MicroTof mass spectrometer (ESI). Infrared spectra were recorded on a Bruker Tensor 27 FT-IR spectrometer as a thin film. Only selected maximum absorbances are reported. Optical rotations were recorded using a Perkin Elmer 341 polarimeter; $[\alpha]_D^T$ values are reported in 10^{-1} deg cm^2 g^{-1}; concentrations (*c*) are quoted in g/100 mL; D refers to the D-line of sodium (589 nm); temperatures (*T*) are given in degrees Celsius (°C). (+) and (−) compound number prefixes indicate the sign of the optical rotation. The enantiomeric excesses were determined by HPLC analysis on an Agilent 1200 Series instrument employing a chiral stationary phase column specified in the individual experiment and by comparing the samples with the appropriate racemic mixtures.

Compound names are those generated by ACD LABS 12.0 following IUPAC nomenclature.

1.4 Starting materials

Catalyst **12**,[1,2] amine **16**,[3] Petasis reagent[4] and nitroolefins **9**[5] and **11**[5] were prepared according to the literature procedures. The following compounds are commercially available: δ-valerolactone (**10**) and hex-5-en-1-amine (**19**).

2 Practical experimental

2.1 Synthesis and characterization of 8

rac-Methyl 2-oxotetrahydro-2*H*-pyran-3-carboxylate (**8**)

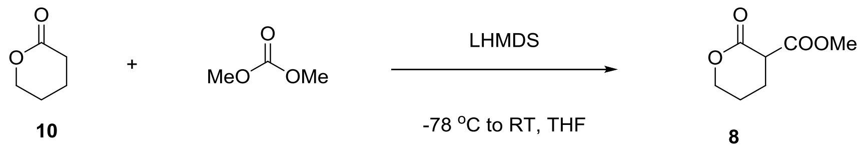

A mixture of δ-valerolactone (**10**, 0.0100 mol, 1.00 g) and dimethyl carbonate (0.011 mol, 0.95 g, 0.88 mL) was added dropwise to a solution of LHMDS in THF (0.0205 mol, 20.5 mL of 1.0 M solution in THF) at −78 °C. The resulting mixture was warmed to rt and stirred at rt. After 4 h the reaction mixture was quenched by the dropwise addition of AcOH (glacial, 1.4 mL) and diluted with Et_2O (60 mL). The resulting suspension was vigorously stirred at rt for 5 min. The insoluble solid was filtered off, washed (Et_2O, 2 × 20 mL) and the filtrate was concentrated in vacuo. The residue was purified by purified by column chromatography (PE:Et_2O 2:1 → 1:1) affording **8** (1.32 g, 83%) as a pale-yellow liquid.

IR (film) 2957 (C-H), 1721 (C=O), 1151 (C-O); **^{1}H-NMR** (400 MHz, $CDCl_3$) δ 1.80 - 1.99 (m, 2H), 2.09 - 2.26 (m, 2H), 3.53 (t, 1H, *J* = 8.0 Hz), 3.73 (s, 3H), 4.20 - 4.35 (m, 2H); **^{13}C-NMR** (100 MHz, $CDCl_3$) δ 20.7, 22.6, 47.0, 52.7, 69.3, 167.3, 169.4; **HRMS** (ES+) exact mass calculated for $[M+Na]^+$ ($C_7H_{10}NaO_4$) requires *m/z* 181.0471, found *m/z* 181.0474.

2.2 Synthesis and characterization of 13 and 14

Methyl (3*S*)-3-[(1*S*)-1-(furan-3-yl)-2-nitroethyl]-2-oxooxane-3-carboxylate (**13**) and methyl (3*R*)-3-[(1*S*)-1-(furan-3-yl)-2-nitroethyl]-2-oxooxane-3-carboxylate (**14**)

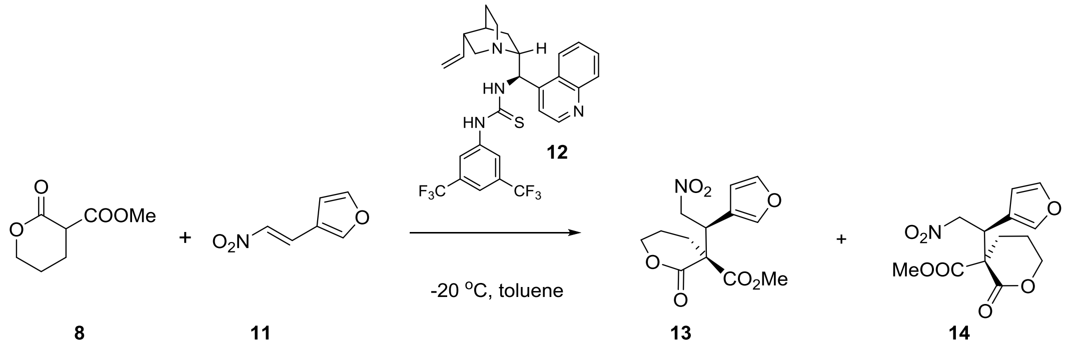

Catalyst **12** (0.040 mmol, 22.6 mg) was added to a solution of pronucleophile **8** (0.22 mmol, 34.8 mg) and electrophile **11** (0.20 mmol, 27.8 mg) in toluene (1.0 mL) at −20 °C. The resulting mixture was stirred at −20 °C. After 24 h AcOH (glacial, 0.20 mmol, 11 µL) was added and the mixture concentrated. The residue (crude dr **13**/**14** ~96:4) was purified by column chromatography (PE/EtOAc/CH_2Cl_2 6:4:1) affording an epimeric mixture of **13**/**14** in 99% yield (59 mg, dr 95:5) as a colourless oil and 90:10 er for the major diastereomer **13** [determined by HPLC, Chiralpak AS-H, hexane/isopropanol = 80:20, 1 mL/min, λ = 220 nm, *t* (major) = 30.97 min, *t* (minor) = 36.00 min].

Characterisation corresponding to a 95:5 epimeric mixture:

IR (film) 1735 (C=O), 1680 (C=O), 1554, 1379 (NO_2); $[\alpha]_D^{25}$ = +14.5 (c = 0.27, $CHCl_3$); **^{1}H NMR** (400 MHz, $CDCl_3$) δ 1.82 - 2.03 (m, 3 H), 2.26 - 2.36 (m, 1 H), 3.74 (s, 3 H), 4.07 - 4.17 (m, 1 H), 4.20 - 4.29 (m, 2 H), 4.72 (dd, *J* = 13.2, 10.8 Hz, 1 H), 5.03 (dd, *J* = 13.2, 3.7 Hz, 1 H), 6.41 (d, *J* = 1.0 Hz, 1 H), 7.33 - 7.43 (m, 2 H); **^{13}C NMR** (100 MHz, $CDCl_3$) δ 20.1, 27.1, 39.8, 53.3, 56.4, 69.0, 76.6, 110.5, 119.1, 142.2, 143.4, 168.3, 169.9; **HRMS** (ES+) exact mass calculated for $[M+Na]^+$ ($C_{13}H_{15}NNaO_7$) requires *m/z* 320.0741, found *m/z* 320.0740.

2.3 Synthesis and characterization of *rac*-13 and *rac*-14

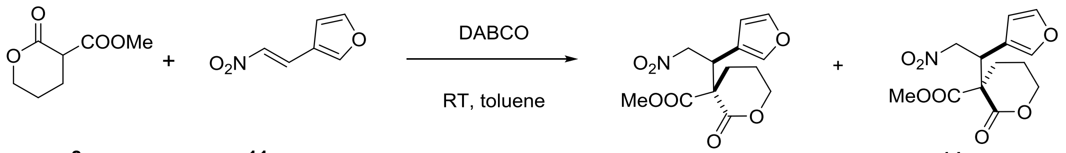

8 **11** **13** **14**

A solution of pronucleophile **8** (0.00400 mol, 0.633 g), electrophile **11** (0.00480 mol, 0.668 g) and DABCO (0.00080 mol, 0.090 g) in toluene (4.0 mL) was stirred at rt. After 1 h another portion of DABCO (0.00040 mol, 0.045 g) was added and the resulting mixture stirred for another 16 h and then it was concentrated in vacuo. The residue (dr **13**/**14** ~45:55) was purified by column chromatography (PE/Et_2O 1:2 → Et_2O, dr **13/14** 57:43) and trituration (Et_2O) affording an epimeric mixture of **13/14** (0.620 g, 52%, dr **13/14** 64:36) as a colourless solid. Iterative recrystallisations afforded crystals of single diastereomer **14** suitable for X-ray analysis.

Characterisation for *rac*-**14**:

M.p. 131-132 °C (EtOH); **IR** (film) 2966, 2941 (C-H), 1729, 1710 (C=O), 1553, 1383 (NO_2); **^{1}H NMR** (400 MHz, $CDCl_3$): δ 1.63 - 1.72 (m, 1 H), 1.83 - 2.02 (m, 2 H), 2.14 - 2.24 (m, 1 H), 3.84 (s, 3 H), 3.96 (dd, 1 H, *J* = 10.5, 2.9 Hz), 3.99 - 4.08 (m, 1 H), 4.28 - 4.38 (m, 1 H), 4.74 (dd, 1 H, *J* = 13.6, 2.8 Hz), 5.15 (dd, 1 H, *J* = 13.7, 10.5 Hz), 6.32 (t, 1 H, *J* = 1.3 Hz), 7.36 - 7.41 (m, 2 H); **^{13}C NMR** (100 MHz, $CDCl_3$) δ 20.2, 30.5, 41.0, 53.6, 57.3, 69.8, 78.4, 109.7, 119.4, 142.3, 144.1, 168.3, 171.1; **HRMS** (ES+) exact mass calculated for $[M+Na]^+$ ($C_{13}H_{15}NNaO_7$) requires *m/z* 320.0741, found *m/z* 320.0743.

For the characterisation of **13** see section 2.2.

2.4 Synthesis and characterisation of 15

Methyl (3*S*,4*S*,5*R*)-1-butyl-4-(furan-3-yl)-3-(3-hydroxypropyl)-5-nitro-2-oxopiperidine-3-carboxylate (**15**)

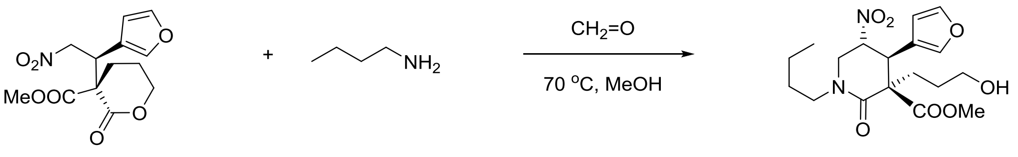

13 **15**

Butylamine (0.30 mmol, 0.022 g, 30 μL) was added to a solution of Michael adduct **13** (59 mg, 0.20 mmol) and formaldehyde (0.30 mmol, 0.0090 g, 22 μL of 37% solution in water) in MeOH (0.4 mL) at rt. The resulting mixture was stirred at reflux. After 1 h the mixture was cooled to rt and concentrated in vacuo. The residue was dissolved in EtOAc and washed with HCl (0.5 M, 2 mL). The separated aqueous phase was extracted with EtOAc (2 × 4 mL). The combined organics were dried (Na_2SO_4) and concentrated in vacuo. The residue was purified by column chromatography (Et_2O) to afford title compound **15** (0.048 g, 63%) as a pale-yellow oil.

IR (film) 3426 (OH), 2956, 2934, 2874 (C-H), 1750, 1640 (C=O), 1556, 1348 (NO_2); $[\alpha]_D^{25}$ = +70.8 (c = 1.2, $CHCl_3$); **^{1}H NMR** (400 MHz, $CDCl_3$) δ 0.95 (t, *J* = 7.5 Hz, 3 H), 1.34 (sxt, *J* = 7.5 Hz, 2 H), 1.40 - 1.74 (m, 4 H), 1.83 (ddd, *J* = 14.0, 11.5, 4.5 Hz, 1 H), 2.01 - 2.07 (m, 1 H), 2.35 (ddd, *J* = 14.0, 11.5, 5.0 Hz, 1 H), 3.36 (ddd, *J* = 13.5, 8.5, 6.5 Hz, 1 H), 3.51 - 3.63 (m, 2 H), 3.65 (s, 3 H), 3.67 - 3.79 (m, 2 H), 3.80 - 3.90 (m, 2 H), 5.75 (ddd, *J* = 12.0, 8.5, 6.5 Hz, 1 H), 6.20 - 6.25 (m, 1 H), 7.32 - 7.37 (m, 2 H); **^{13}C NMR** (100 MHz, $CDCl_3$) δ 13.7, 19.9, 27.0, 28.3, 28.8, 39.5, 48.0, 49.5, 52.8, 58.4, 62.1, 82.0, 109.1, 118.0, 141.4, 143.7, 166.3, 170.8; **HRMS** (ES+) exact mass calculated for $[M+Na]^+$ ($C_{18}H_{26}N_2NaO_7$) requires *m/z* 405.1637, found *m/z* 405.1632.

2.5 Synthesis and characterisation of 7 and 7’

Methyl (3*S*)-3-[(1*S*)-2-nitro-1-[5-(pent-3-yn-1-yl)furan-3-yl]ethyl]-2-oxooxane-3-carboxylate (**7**) and methyl (3*R*)-3-[(1*S*)-2-nitro-1-[5-(pent-3-yn-1-yl)furan-3-yl]ethyl]-2-oxooxane-3-carboxylate (**7’**)

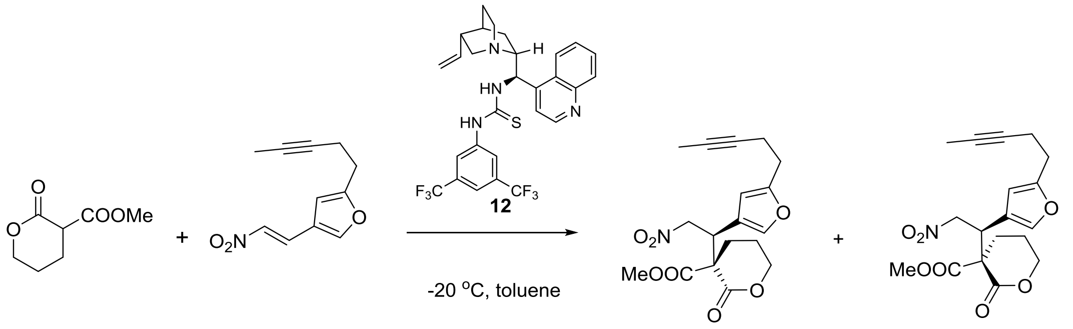

Catalyst **12** (0.16 mmol, 0.090 g) was added to a solution of pronucleophile **8** (0.880 mmol, 0.139 g) and electrophile **9** (0.800 mmol, 0.165 g) in toluene (4.0 mL) at −20 °C. The resulting mixture was stirred at −20 °C. After 36 h AcOH (glacial, 0.80 mmol, 0.048 g, 46 µL) was added and the mixture was concentrated in vacuo. The residue (crude dr **7**/**7’** ~95:5) was purified by column chromatography (PE/EtOAc/CH_2Cl_2 6:4:1) affording an epimeric mixture of **7**/**7’** (0.268 g, dr 95:5) as a pale-yellow oil in 92% yield.[a]

^{1}H NMR (400 MHz, $CDCl_3$) δ 1.76 (t, *J* = 2.5 Hz, 3 H), 1.83 - 2.07 (m, 3 H) 2.27 - 2.36 (m, 1 H), 2.42 (tq, *J* = 7.4, 2.5 Hz, 2 H), 2.74 (t, *J* = 7.3 Hz, 2 H), 3.77 (s, 3 H), 4.12 - 4.30 (m, 3 H), 4.71 (dd, *J* = 13.1, 10.6 Hz, 1 H), 5.03 (dd, *J* = 13.2, 3.7 Hz, 1 H), 6.11 (s, 1 H) 7.26 (s, 1 H); **^{13}C NMR** (100 MHz, $CDCl_3$) δ 3.5, 17.9, 20.4, 27.1, 28.0, 40.1, 53.4, 56.6, 69.1, 76.6, 76.7, 77.7, 106.6, 119.7, 140.8, 155.7, 168.5, 170.1; **HRMS** (ES+) exact mass calculated for $[M+Na]^+$ ($C_{18}H_{21}NNaO_7$) requires *m/z* 386.1210, found *m/z* 386.1209.

Minor diastereomer 7’ (observable)

^{1}H NMR (400 MHz, $CDCl_3$) δ 3.86 (s, 3 H), 3.92 (dd, *J* = 10.5, 2.7 Hz, 1 H), 4.07 (m, *J* = 3.7 Hz, 1 H), 4.34 - 4.42 (m, 1 H), 5.14 (dd, *J* = 13.5, 10.5 Hz, 1 H), 6.03 (s, 1 H); **^{13}C NMR** (100 MHz, $CDCl_3$) δ 41.4, 53.7, 70.0, 78.6, 105.7, 140.5.

[a] The enantiomeric ratio was determined on compound **6**.

2.6 Synthesis and characterisation of 6

Methyl (3*R*,4*S*,5*R*)-1-(hept-5-yn-1-yl)-3-(3-hydroxypropyl)-5-nitro-2-oxo-4-[5-(pent-3-yn-1-yl)furan-3-yl]piperidine-3-carboxylate (**6**)

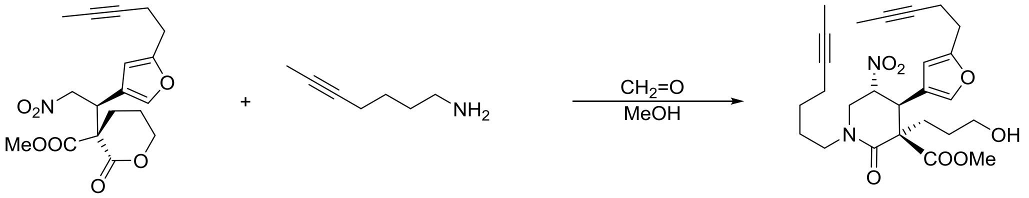

7 **16** **6**

Hept-5-yn-1-amine (**16**, 1.20 mmol, 0.133 g) was added to a solution of Michael adduct **7** (267 mg, 0.736 mmol,) and formaldehyde (1.2 mmol, 0.036 g, 89 µL of 37% solution in water) and MeOH (1.6 mL) at rt. The resulting mixture was stirred at reflux. After 1 h the mixture was cooled to rt and concentrated in vacuo. The residue was dissolved in EtOAc (8 mL) and washed with HCl (0.5 M, 8 mL). The separated aqueous phase was extracted with EtOAc (2 × 8 mL). The combined organics were dried (Na_2SO_4) and concentrated in vacuo. The residue was purified by column chromatography (Et_2O) to afford title compound **6** (0.201 g, 56%) as a yellow oil and 91:9 er [determined by HPLC, Chiralpak OD-H, hexane/isopropanol 50:50, 0.6 mL/min, λ = 220 nm, *t* (major) = 11.81 min, *t* (minor) = 15.21 min].

IR (film) 3443 (OH), 2920, 2860 (C-H), 1752, 1646 (C=O), 1557, 1374 (NO_2); $[\alpha]_D^{25}$ = +47.8 (c = 3.9, $CHCl_3$); **^{1}H NMR** (400 MHz, $CDCl_3$) δ 1.36 - 1.53 (m, 3 H), 1.62 - 1.87 (m, 9 H), 1.95 (br. s, 1 H), 2.10 - 2.22 (m, 2 H), 2.26 - 2.43 (m, 3 H), 2.70 (t, *J* = 7.3 Hz, 2 H), 3.35 - 3.46 (m, 1 H), 3.48 - 3.74 (m, 7 H), 3.77 - 3.88 (m, 2 H), 5.71 (ddd, *J* = 12.0 Hz, 9.2 Hz, 6.1 Hz, 1 H), 5.83 - 5.90 (m, 1 H), 7.14 - 7.20 (m, 1 H); **^{13}C NMR** (100 MHz, $CDCl_3$) δ 3.4, 3.5, 17.6, 18.3, 25.8, 25.9, 27.2, 27.8, 28.3, 39.8, 47.6, 49.5, 52.7, 58.4, 62.2, 76.2, 76.6, 77.5, 78.4, 82.0, 105.0, 118.4, 139.9, 155.9, 166.6, 170.8; **HRMS** (ES+) exact mass calculated for $[M+Na]^+$ ($C_{26}H_{34}N_2NaO_7$) requires *m/z* 509.2258, found *m/z* 509.2265.

2.7 Synthesis of *rac*-6

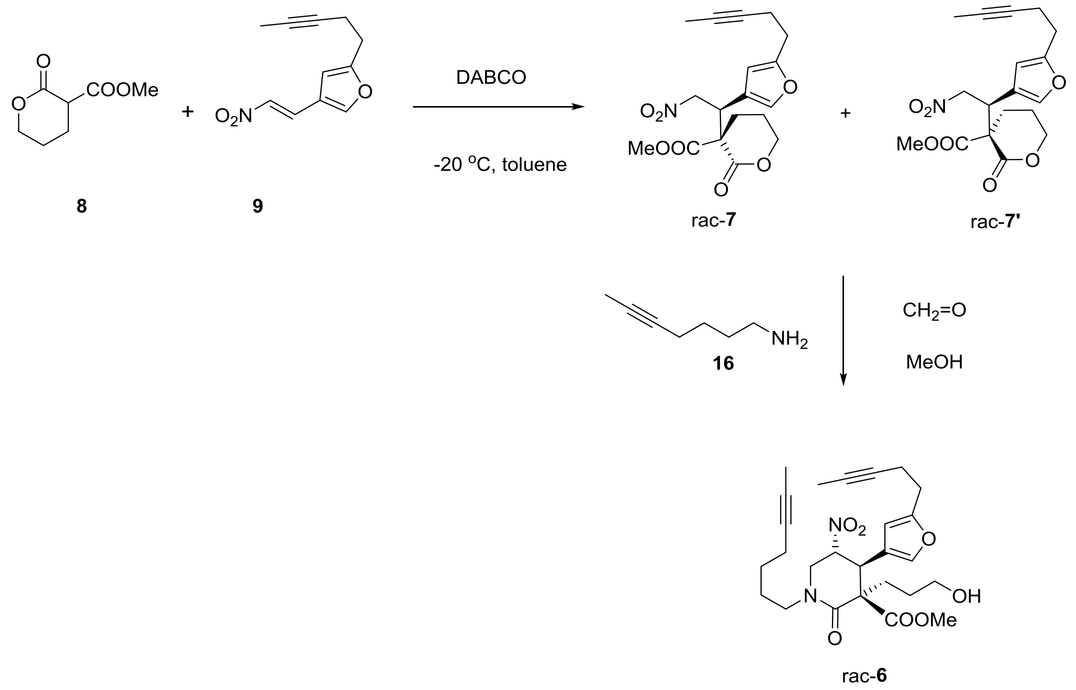

A solution of pronucleophile **8** (2.20 mol, 0.348 g), electrophile **9** (2.00 mmol, 0.410 g) and DABCO (1.00 mol, 0.112 g) in toluene (2.0 mL) was stirred at rt with exclusion of light. After 24 h another portion of DABCO (1.00 mmol, 0.112 g) was added and the resulting mixture stirred for another 24 h before being concentrated in vacuo. The residue (dr **7**/**7'** ~36:64) was purified by column chromatography (PE/Et_2O 1:2 → Et_2O), affording an brown oil (0.136 g) containing an epimeric mixture of **7**/**7'** (dr ~50:50). This mixture was dissolved in MeOH (0.7 mL), and formaldehyde (0.54 mmol, 0.16 g, 40 µL of 37% aqueous solution) and hept-5-yn-1-amine (**16**, 0.54 mmol, 0.060 g) were added at rt. The resulting solution was stirred at reflux. After 1 h the mixture was cooled to rt and concentrated in vacuo. The residue was dissolved in EtOAc (8 mL) and washed with HCl (0.5 M, 8 mL). The separated aqueous phase was extracted with EtOAc (2 × 8 mL). The combined organics were dried (Na_2SO_4) and concentrated in vacuo. The residue was purified by column chromatography (Et_2O) to afford title compound *rac*-**6** (0.045 g, 5% over 2 steps from nitroolefin **9**) as a yellow oil.

Characterisation data for *rac*-**6** are consistent with that given for **6** (section 2.6).

2.8 Synthesis and characterization of 17

Methyl (3*R*,4*S*)-1-(hept-5-yn-1-yl)-3-(3-hydroxypropyl)-2-oxo-4-[5-(pent-3-yn-1-yl)furan-3-yl]piperidine-3-carboxylate (**17**)

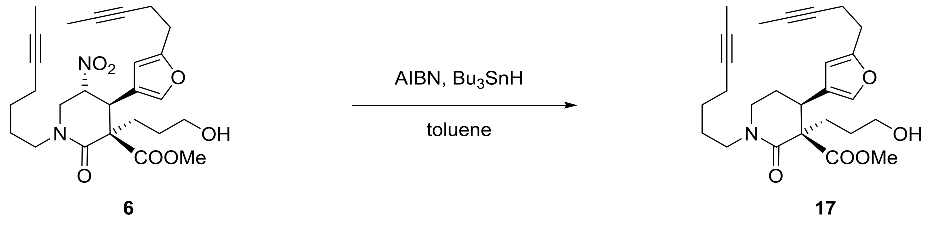

To a solution of spirocycle **6** (0.164 mmol, 0.0800 g) in toluene (6.6 mL) was added Bu_3SnH (0.82 mmol, 0.24 g, 0.22 mL) and AIBN (0.082 mmol, 0.013 g) under an atmosphere of Ar. The resulting mixture was degassed and filled with N_2 (this operation was repeated 5 times). The suspension was stirred at reflux for 20 min then cooled to rt and concentrated in vacuo. The residue was purified by column chromatography (MTBE/PE 3:1 → MTBE) to afford title compound **17** (0.051 g, 71%) as a pale-yellow oil.

IR (film) 3420 (OH), 2920, 2861 (C-H), 1744 (C=O), 1621 (C=O); $[\alpha]_D^{25}$ = +63.3 (c = 2.4, $CHCl_3$); **^{1}H NMR** (400 MHz, $CDCl_3$) δ 1.31 - 1.54 (m, 3 H), 1.60 - 1.79 (m, 9 H), 1.80 - 1.94 (m, 2 H), 2.06 (br. s., 1 H), 2.12 - 2.31 (m, 3 H), 2.39 (tq, *J* = 7.4, 2.4 Hz, 2 H), 2.61 (dq, *J* = 12.3, 5.5 Hz, 1 H), 2.72 (t, *J* = 7.5 Hz, 2 H), 3.05 (dd, *J* = 13.3, 3.1 Hz, 1 H), 3.31 - 3.63 (m, 8 H), 3.63 - 3.71 (m, 1 H), 5.86 (s, 1 H), 7.10 (s, 1 H); **^{13}C NMR** (100 MHz, $CDCl_3$) δ 3.40, 3.41, 17.8, 18.4, 25.3, 26.0, 26.2, 27.3, 27.9, 28.7, 35.7, 47.2, 47.4, 51.9, 58.7, 62.4, 75.8, 76.3, 77.8, 78.8, 105.6, 124.4, 138.0, 155.0, 168.4, 171.7; **HRMS** (ES+) exact mass calculated for $[M+Na]^+$ ($C_{26}H_{35}NNaO_5$) requires *m/z* 464.2407, found *m/z* 464.2405.

2.9 Synthesis and characterisation of 18

(6*R*,11*S*)-8-(Hept-5-yn-1-yl)-11-[5-(pent-3-yn-1-yl)furan-3-yl]-2-oxa-8-azaspiro[5.5]undecane-1,7-dione (**18**)

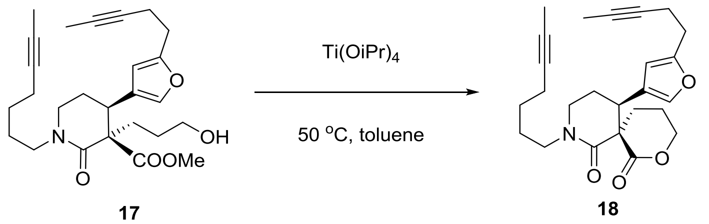

$Ti(OiPr)_4$ (1.1 mmol, 0.31 g, 0.30 mL) was added dropwise to a solution of ester **17** (1.00 mmol, 0.441 g) in toluene (10 mL) at rt and the resulting solution was stirred at 50 °C. After 2 h the mixture was cooled to rt, concentrated in vacuo and the residue was filtered through a plug of silica gel eluted with Et_2O. The filtrate was concentrated in vacuo and the residue was purified by column chromatography (PE/Et_2O 1:2 →Et_2O) to afford **18** (0.344 g, 84%) as a pale-yellow oil.

IR (film) 2920, 2860 (C-H), 1716, 1631 (C=O); $[\alpha]_D^{25}$ = +70.9 (c = 2.7, $CHCl_3$); **^{1}H NMR** (400 MHz, $CDCl_3$) δ 1.46 - 1.60 (m, 2 H), 1.64 - 1.79 (m, 10 H), 1.83 - 1.97 (m, 1 H), 2.10 - 2.22 (m, 2 H), 2.28 - 2.47 (m, 4 H), 2.71 - 2.83 (m, 3 H), 2.89 - 3.07 (m, 1 H), 3.32 - 3.52 (m, 4 H), 3.72 (dt, *J* = 11.0 Hz, 2.2 Hz, 1 H), 4.22 - 4.34 (m, 1 H), 6.06 (s, 1 H) 7.22 (s, 1 H); **^{13}C NMR** (100 MHz, $CDCl_3$) 3.36, 3.42, 17.8, 18.4, 22.7, 25.6, 26.0, 26.1, 27.9, 32.9, 42.5, 47.3, 47.4, 53.5, 70.0, 75.7, 76.3, 77.7, 78.7, 106.0, 124.1, 138.7 155.6, 169.6, 170.2; **HRMS** (ES+) exact mass calculated for $[M+Na]^+$ ($C_{25}H_{31}NNaO_4$) requires *m/z* 432.2145, found *m/z* 432.2152.

2.10 Synthesis and characterisation of 20

(3*S*,4*S*)-1-(Hept-5-yn-1-yl)-*N*-(hex-5-en-1-yl)-3-(3-hydroxypropyl)-2-oxo-4-[5-(pent-3-yn-1-yl)furan-3-yl]piperidine-3-carboxamide (**20**)

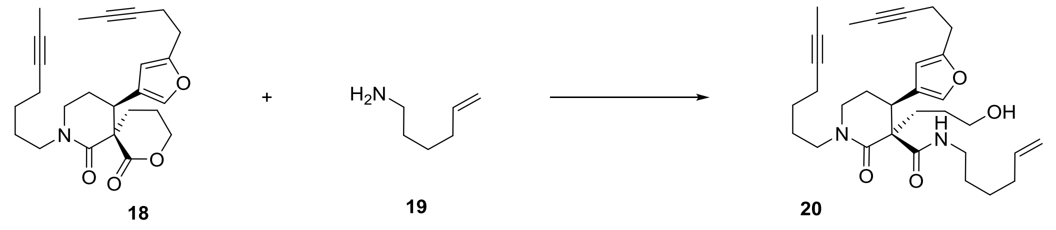

A mixture of lactone **18** (0.195 mmol, 0.0800 g) and amine **19** (0.78 mmol, 0.077 g) was stirred at 130 °C. After 24 h the mixture was cooled to rt and purified by column chromatography (EtOAc) to afford amide **20** (0.066 g, 67%) as a pale-yellow oil.

IR (film) 3379 (OH, NH), 2921, 2859 (C-H), 1654 (C=O); $[\alpha]_D^{25}$ = -13.8 (c = 1.0, $CHCl_3$); **^{1}H NMR** (400 MHz, $CDCl_3$) δ 1.23 - 1.72 (m, 10 H), 1.73 - 1.79 (m, 6 H), 1.82 - 2.20 (m, 8 H), 2.24 - 2.43 (m, 3 H), 2.71 (t, *J* = 7.7 Hz, 2 H), 3.03 - 3.23 (m, 3 H), 3.30 (dd, *J* = 8.2, 5.0 Hz, 2 H), 3.44 (t, *J* = 4.5 Hz, 1 H), 3.49 - 3.65 (m, 3 H), 4.90 - 5.03 (m, 2 H), 5.75 (ddt, *J* = 17.1, 10.2, 6.6 Hz, 1 H), 5.89 (s, 1 H), 7.05 (s, 1 H), 8.32 (br. s., 1 H); **^{13}C NMR** (100 Hz, $CDCl_3$) δ 3.41, 3.43, 17.8, 18.4, 23.8, 25.8, 26.2, 26.3, 28.0, 28.4, 28.7, 33.3, 34.7, 37.0, 39.0, 45.5, 47.2, 54.7, 62.4, 75.9, 76.1, 77.9, 78.7, 106.4, 114.6, 125.2, 137.7, 138.5, 154.5, 170.9, 172.2; **HRMS** (ES+) exact mass calculated for $[M+Na]^+$ ($C_{31}H_{44}N_2NaO_4$) requires *m/z* 531.3193, found *m/z* 531.3204.

2.11 Synthesis and characterisation of 21

(3*S*,4*S*)-1-(Hept-5-yn-1-yl)-*N*-(hex-5-en-1-yl)-2-oxo-3-(3-oxopropyl)-4-[5-(pent-3-yn-1-yl)furan-3-yl]piperidine-3-carboxamide (**21**)

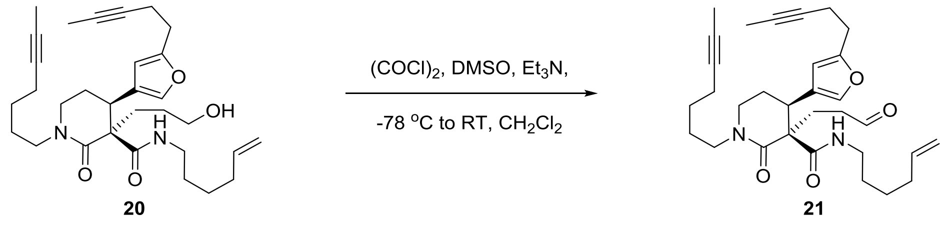

To a solution of oxalyl chloride (0.083 mmol, 0.010 g, 8 µL) in CH_2Cl_2 (4 mL) was added dropwise DMSO (0.17 mmol, 0.013 g, 12 µL) at −78 °C and the mixture was stirred at −78 °C. After 3 min a solution of alcohol **20** (0. 079 mmol, 0.040 g) in CH_2Cl_2 (1 mL) was added dropwise and the solution was stirred at −78 °C. After 15 min Et_3N (5 equiv, 0.39 mmol, 0.040 g, 55 µL) was added dropwise. The resulting mixture was stirred for 5 min at −78 °C and warmed to rt over 30 min. The mixture was concentrated by a stream of nitrogen and the residue was purified by column chromatography (Et_2O) to afford the title compound **21** (0.035 g, 88%) as a pale-yellow oil.

IR (film) 2920, 2858 (C-H), 1636 (C=O), 1493, 1433; **[*α*]$_D^{24}$** = +40.7 (c = 0.27, $CHCl_3$); **^{1}H NMR** (400 MHz, $CDCl_3$) δ 1.18 - 1.44 (m, 6 H), 1.51 - 1.65 (m, 2 H), 1.66 - 1.72 (m, 6 H), 1.76 - 1.89 (m, 1 H), 1.96 (q, *J* = 7.1 Hz, 2 H), 2.06 - 2.24 (m, 4 H), 2.27 - 2.37 (m, 3 H), 2.40 - 2.54 (m, 1 H), 2.57 - 2.72 (m, 3 H), 3.01 - 3.12 (m, 2 H), 3.14 - 3.29 (m, 3 H), 3.30 - 3.43 (m, 2 H), 4.86 - 4.95 (m, 2 H), 5.70 (ddt, *J* = 17.0, 10.3, 6.8 Hz, 1 H), 5.79 - 5.83 (m, 1 H), 6.97 (s, 1 H), 8.52 (t, *J* = 5.5 Hz, 1 H), 9.66 (s, 1 H); **^{13}C NMR** (100 MHz, $CDCl_3$) δ 3.4, 3.4, 17.7, 18.4, 23.7, 25.7, 26.2, 26.3, 27.9, 28.7, 30.9, 33.2, 37.3, 39.0, 40.1, 45.2, 47.4, 53.6, 76.0, 76.1, 77.9, 78.4, 106.4, 114.7, 125.0, 137.6, 138.4, 154.6, 170.2, 172.0, 200.9; **HRMS** (ES+) exact mass calculated for $[M+Na]^+$ ($C_{31}H_{42}N_2NaO_4$) requires *m/z* 529.3037, found *m/z* 529.3047.

2.12 Synthesis and characterisation of 5

(3*S*,4*S*)-3-(But-3-en-1-yl)-1-(hept-5-yn-1-yl)-*N*-(hex-5-en-1-yl)-2-oxo-4-[5-(pent-3-yn-1-yl)furan-3-yl]piperidine-3-carboxamide (**5**)

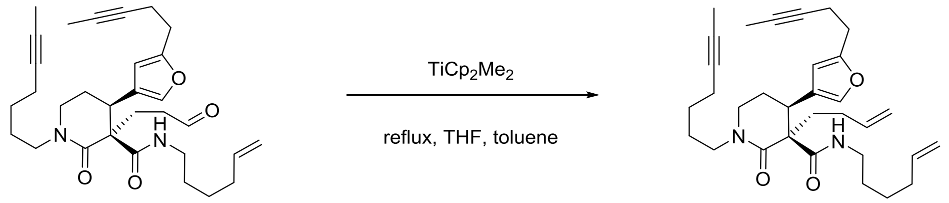

21 **5**

A solution of Petasis reagent[4] (0.21 mmol, 0.45 g of 9.7% solution in THF/toluene) was added to a solution of aldehyde **21** (0.069 mmol, 0.035 g) in toluene (3.5 mL) at rt. The resulting mixture was stirred at reflux. After 20 min, the mixture was cooled to rt and quenched by addition of NH_4Cl (saturated aqueous solution, 4 mL). The resulting emulsion was extracted (Et_2O, 3 × 5 mL), the combined organics were dried (Na_2SO_4) and concentrated in vacuo. The residue was purified by column chromatography (PE/Et_2O 1:2) to afford **5** (0.0146 g, 42%) as a pale-yellow oil.

IR (film) 3282 (N-H), 2922, 2858 (C-H), 1666, 1640 (C=O); $[\alpha]_D^{23}$ = -26 (c = 0.40, $CHCl_3$); **^{1}H NMR** (500 MHz, $CDCl_3$) δ 1.29 - 1.51 (m, 6 H), 1.58 - 1.71 (m, 2 H), 1.74 - 1.80 (m, 6 H), 1.79 - 1.87 (m, 1 H), 1.91 - 2.12 (m, 5 H), 2.13 - 2.20 (m, 3 H), 2.24 - 2.35 (m, 1 H), 2.36 - 2.44 (m, 2 H), 2.72 (t, *J* = 7.6 Hz, 2 H), 3.08 - 3.25 (m, 3 H), 3.29 (dd, *J* = 8.5, 4.6 Hz, 2 H), 3.47 - 3.59 (m, 2 H), 4.91 - 5.05 (m, 4 H), 5.71 - 5.83 (m, 2 H), 5.90 (s, 1 H), 7.05 (s, 1 H), 8.58 (br. s., 1 H); **^{13}C NMR** (100 MHz, $CDCl_3$) δ 3.5, 3.5, 17.8, 18.5, 23.8, 25.9, 26.2, 26.3, 28.0, 28.7, 29.4, 33.3, 36.9, 38.3, 39.0, 45.4, 47.3, 54.4, 75.9, 76.1, 78.0, 78.6, 106.5, 114.6, 115.0, 125.4, 137.6, 137.8, 138.5, 153.9, 170.6, 172.3; **HRMS** (ES+) exact mass calculated for $[M+Na]^+$ ($C_{32}H_{44}N_2NaO_3$) requires *m/z* 527.3244, found *m/z* 527.3253.

3 Single X-ray crystal diffraction data of 14

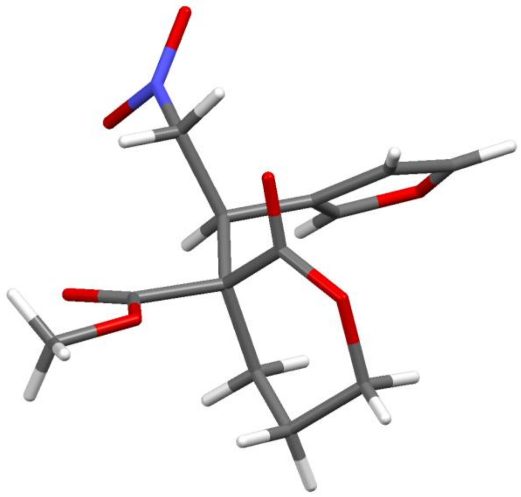

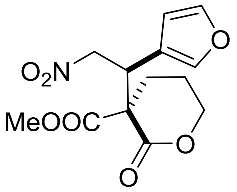

14

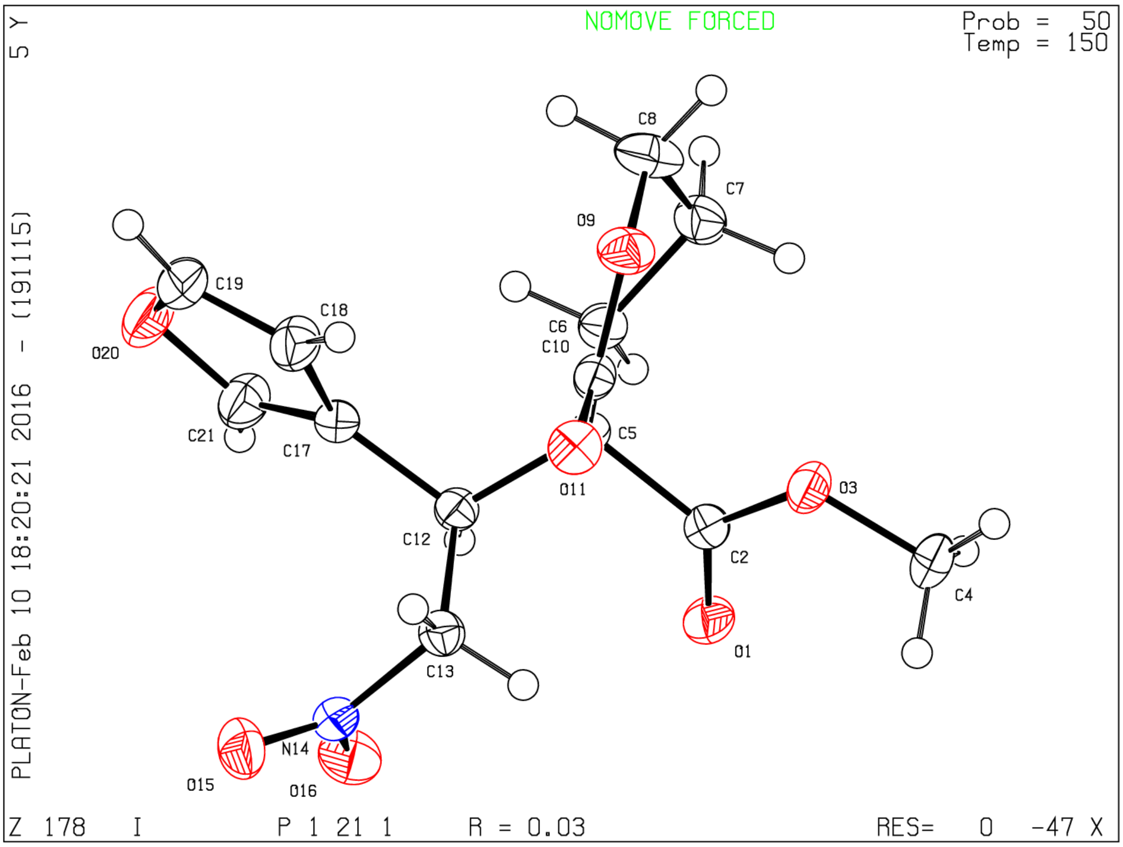

Crystal data

$C_{13}H_{15}NO_7$	Z = 2
M_r = 297.26	F(000) = 312
Monoclinic, $P2_1$	D_x = 1.433 Mg m^{-3}
a = 9.4573 (3) Å	Mo $K\alpha$ radiation, λ = 0.71073 Å
b = 6.5026 (2) Å	Cell parameters from 1362 reflections
c = 11.6625 (4) Å	θ = 5–27°
β = 106.1747 (13)°	μ = 0.12 mm^{-1}
V = 688.82 (4) Å^3	T = 150 K

Data collection

Nonius KappaCCD diffractometer	1629 reflections with $I > 2.0\sigma(I)$
Graphite monochromator	R_{int} = 0.017
ω scans	θ_{max} = 27.5°, θ_{min} = 5.3°
Absorption correction: multi-scan *DENZO*/*SCALEPACK* (Otwinowski & Minor, 1997)	h = −12→12
T_{min} = 1.00, T_{max} = 1.00	k = −8→8
2908 measured reflections	l = −15→15
1706 independent reflections	

Refinement

Refinement on F^2	Primary atom site location: structure-invariant direct methods
Least-squares matrix: full	Hydrogen site location: difference Fourier map
$R[F^2 > 2\sigma(F^2)]$ = 0.030	H-atom parameters constrained
$wR(F^2)$ = 0.071	Method = Modified Sheldrick $w = 1/[\sigma^2(F^2) + (0.03P)^2 + 0.19P]$, where $P = (\max(F_o^2,0) + 2F_c^2)/3$
S = 0.97	$(\Delta/\sigma)_{max}$ = 0.0001
1706 reflections	$\Delta\rho_{max}$ = 0.21 e Å^{-3}
190 parameters	$\Delta\rho_{min}$ = −0.16 e Å^{-3}
1 restraint	

Experimental

The crystal was placed in the cold stream of an Oxford Cryosystems open-flow nitrogen cryostat (Cosier & Glazer, 1986) with a nominal stability of 0.1 K.[6]

Fractional atomic coordinates and isotropic or equivalent isotropic displacement parameters (Å^2)

	x	y	z	U_{iso}*/U_{eq}
O1	−0.08721 (13)	0.7478 (2)	0.05969 (10)	0.0273
C2	−0.04558 (18)	0.7324 (3)	0.16680 (15)	0.0210
O3	−0.12390 (12)	0.7872 (2)	0.24067 (11)	0.0271
C4	−0.27248 (18)	0.8633 (3)	0.18546 (17)	0.0312
C5	0.10166 (17)	0.6356 (3)	0.23553 (14)	0.0193
C6	0.07400 (19)	0.4012 (3)	0.23895 (16)	0.0237
C7	0.0032 (2)	0.3474 (3)	0.33667 (17)	0.0302
C8	0.1065 (3)	0.4121 (4)	0.45394 (18)	0.0378
O9	0.14496 (14)	0.6312 (2)	0.45674 (11)	0.0295
C10	0.14760 (18)	0.7358 (3)	0.35917 (15)	0.0217
O11	0.18367 (13)	0.9148 (2)	0.37052 (11)	0.0270
C12	0.22014 (18)	0.6745 (3)	0.16807 (15)	0.0203
C13	0.23838 (19)	0.9012 (3)	0.13928 (15)	0.0233
N14	0.31655 (15)	0.9119 (3)	0.04381 (13)	0.0245
O15	0.43140 (14)	1.0090 (3)	0.06451 (13)	0.0372
O16	0.26083 (15)	0.8250 (3)	−0.05085 (11)	0.0360
C17	0.36734 (18)	0.5822 (3)	0.23222 (15)	0.0226
C18	0.4593 (2)	0.6208 (4)	0.35131 (17)	0.0317
C19	0.5787 (2)	0.5008 (4)	0.36549 (17)	0.0350
O20	0.57129 (15)	0.3908 (3)	0.26536 (13)	0.0391
C21	0.4410 (2)	0.4433 (3)	0.18530 (17)	0.0319
H41	−0.3131	0.9006	0.2491	0.0478*
H43	−0.2672	0.9837	0.1381	0.0484*
H42	−0.3312	0.7534	0.1363	0.0486*
H61	0.1696	0.3313	0.2539	0.0291*
H62	0.0102	0.3598	0.1618	0.0288*
H72	−0.0931	0.4174	0.3234	0.0342*
H71	−0.0090	0.1962	0.3388	0.0345*
H81	0.0627	0.3881	0.5187	0.0461*
H82	0.2017	0.3342	0.4706	0.0465*
H121	0.1831	0.6014	0.0901	0.0250*
H131	0.2996	0.9722	0.2091	0.0290*
H132	0.1425	0.9658	0.1068	0.0295*
H181	0.4387	0.7139	0.4080	0.0388*
H191	0.6626	0.4905	0.4348	0.0425*
H211	0.4136	0.3846	0.1092	0.0395*

Atomic displacement parameters (Å^2)

	U^{11}	U^{22}	U^{33}	U^{12}	U^{13}	U^{23}
O1	0.0254 (6)	0.0343 (8)	0.0196 (6)	0.0020 (6)	0.0019 (5)	0.0022 (6)

C2	0.0211 (7)	0.0197 (8)	0.0215 (8)	−0.0015 (7)	0.0046 (6)	−0.0011 (7)
O3	0.0215 (6)	0.0361 (8)	0.0231 (6)	0.0083 (6)	0.0053 (5)	0.0009 (6)
C4	0.0199 (8)	0.0373 (12)	0.0351 (9)	0.0078 (8)	0.0052 (7)	0.0043 (9)
C5	0.0198 (7)	0.0200 (8)	0.0173 (7)	0.0015 (7)	0.0041 (6)	0.0002 (7)
C6	0.0265 (8)	0.0206 (8)	0.0243 (8)	0.0010 (7)	0.0077 (6)	−0.0002 (7)
C7	0.0316 (9)	0.0241 (9)	0.0376 (10)	0.0001 (8)	0.0139 (7)	0.0051 (8)
C8	0.0547 (12)	0.0336 (11)	0.0282 (10)	0.0002 (10)	0.0168 (9)	0.0089 (9)
O9	0.0364 (7)	0.0334 (8)	0.0192 (6)	0.0017 (6)	0.0086 (5)	0.0020 (6)
C10	0.0192 (7)	0.0267 (9)	0.0189 (8)	0.0034 (7)	0.0049 (6)	−0.0004 (7)
O11	0.0301 (6)	0.0254 (7)	0.0249 (6)	−0.0009 (6)	0.0064 (5)	−0.0056 (6)
C12	0.0218 (8)	0.0225 (9)	0.0171 (7)	0.0015 (7)	0.0061 (6)	−0.0004 (7)
C13	0.0244 (8)	0.0234 (9)	0.0232 (8)	0.0015 (7)	0.0086 (6)	0.0006 (7)
N14	0.0239 (7)	0.0238 (8)	0.0266 (7)	0.0050 (7)	0.0082 (6)	0.0053 (7)
O15	0.0249 (6)	0.0382 (8)	0.0506 (8)	−0.0029 (6)	0.0139 (6)	0.0043 (7)
O16	0.0443 (8)	0.0408 (9)	0.0243 (6)	−0.0006 (7)	0.0120 (6)	−0.0017 (7)
C17	0.0219 (7)	0.0255 (9)	0.0206 (8)	0.0015 (7)	0.0062 (6)	0.0035 (7)
C18	0.0265 (9)	0.0442 (12)	0.0231 (9)	0.0046 (9)	0.0050 (7)	0.0005 (9)
C19	0.0257 (9)	0.0469 (13)	0.0307 (9)	0.0065 (9)	0.0052 (7)	0.0099 (10)
O20	0.0309 (7)	0.0458 (10)	0.0399 (8)	0.0171 (7)	0.0086 (6)	0.0042 (8)
C21	0.0296 (9)	0.0353 (11)	0.0301 (9)	0.0082 (9)	0.0074 (7)	−0.0002 (9)

Geometric parameters (Å, º)

O1—C2	1.205 (2)	O9—C10	1.332 (2)
C2—O3	1.332 (2)	C10—O11	1.210 (2)
C2—C5	1.535 (2)	C12—C13	1.533 (3)
O3—C4	1.459 (2)	C12—C17	1.509 (2)
C4—H41	0.957	C12—H121	0.999
C4—H43	0.967	C13—N14	1.500 (2)
C4—H42	0.985	C13—H131	0.974
C5—C6	1.549 (3)	C13—H132	0.975
C5—C10	1.531 (2)	N14—O15	1.221 (2)
C5—C12	1.559 (2)	N14—O16	1.222 (2)
C6—C7	1.515 (3)	C17—C18	1.441 (2)
C6—H61	0.983	C17—C21	1.346 (3)
C6—H62	0.972	C18—C19	1.344 (3)
C7—C8	1.503 (3)	C18—H181	0.956
C7—H72	0.991	C19—O20	1.355 (3)
C7—H71	0.991	C19—H191	0.965
C8—O9	1.468 (3)	O20—C21	1.366 (2)
C8—H81	0.970	C21—H211	0.934
C8—H82	1.004		
O1—C2—O3	124.63 (15)	H81—C8—H82	108.8

O1—C2—C5	124.24 (15)	C8—O9—C10	122.69 (16)
O3—C2—C5	111.05 (13)	C5—C10—O9	121.29 (16)
C2—O3—C4	116.49 (13)	C5—C10—O11	120.66 (17)
O3—C4—H41	106.8	O9—C10—O11	118.02 (17)
O3—C4—H43	109.3	C5—C12—C13	114.02 (14)
H41—C4—H43	109.5	C5—C12—C17	112.27 (14)
O3—C4—H42	109.2	C13—C12—C17	110.28 (15)
H41—C4—H42	110.6	C5—C12—H121	105.3
H43—C4—H42	111.4	C13—C12—H121	106.9
C2—C5—C6	106.36 (14)	C17—C12—H121	107.6
C2—C5—C10	107.20 (13)	C12—C13—N14	108.44 (14)
C6—C5—C10	113.59 (15)	C12—C13—H131	110.4
C2—C5—C12	110.19 (13)	N14—C13—H131	107.3
C6—C5—C12	108.83 (14)	C12—C13—H132	110.5
C10—C5—C12	110.56 (13)	N14—C13—H132	106.8
C5—C6—C7	111.09 (16)	H131—C13—H132	113.1
C5—C6—H61	107.8	C13—N14—O15	118.21 (15)
C7—C6—H61	109.7	C13—N14—O16	118.14 (15)
C5—C6—H62	108.3	O15—N14—O16	123.65 (15)
C7—C6—H62	110.0	C12—C17—C18	129.38 (16)
H61—C6—H62	109.8	C12—C17—C21	125.14 (16)
C6—C7—C8	107.97 (16)	C18—C17—C21	105.47 (16)
C6—C7—H72	110.8	C17—C18—C19	106.15 (18)
C8—C7—H72	110.5	C17—C18—H181	126.3
C6—C7—H71	109.1	C19—C18—H181	127.5
C8—C7—H71	107.8	C18—C19—O20	111.16 (16)
H72—C7—H71	110.6	C18—C19—H191	127.6
C7—C8—O9	112.73 (17)	O20—C19—H191	121.2
C7—C8—H81	110.7	C19—O20—C21	106.10 (15)
O9—C8—H81	107.2	O20—C21—C17	111.12 (17)
C7—C8—H82	110.8	O20—C21—H211	120.4
O9—C8—H82	106.4	C17—C21—H211	128.5

Hydrogen-bond geometry (Å, º)

D—H···*A*	*D*—H	H···*A*	*D*···*A*	*D*—H···*A*
C4—H43···O16^{i}	0.97	2.45	3.404 (3)	169 (1)
C6—H62···O16^{ii}	0.97	2.54	3.349 (3)	141 (1)
C7—H71···O11^{iii}	0.99	2.54	3.256 (3)	130 (1)
C19—H191···O11^{iv}	0.97	2.38	3.315 (3)	163 (1)
C21—H211···O15^{iii}	0.93	2.51	3.146 (3)	125 (1)

Symmetry codes: (i) −*x*, *y*+1/2, −*z*; (ii) −*x*, *y*−1/2, −*z*; (iii) *x*, *y*−1, *z*; (iv) −*x*+1, *y*−1/2, −*z*+1.

4 References

(1) Manna, M. S.; Kumar, V.; Mukherjee, S. *Chem. Commun.* **2012**, *48*, 5193-5195.

(2) Ye, J.; Dixon, D. J.; Hynes, P. S. *Chem. Commun.* **2005**, 4481-4483.

(3) Jakubec, P.; Kyle, A. F.; Calleja, J.; Dixon, D. J. *Tetrahedron Lett.* **2011**, *52*, 6094-6097.

(4) Payack, J. F.; Hughes, D. L.; Cai, D.; Cottrell, I. F.; Verhoeven, T. R. *Org. Synth.* **2002**, *79*, 19-23.

(5) Kyle, A. F.; Jakubec, P.; Cockfield, D. M.; Cleator, E.; Skidmore, J.; Dixon, D. J. *Chem. Commun.* **2011**, *47*, 10037-10039.

(6) Cosier, J.; Glazer, A. M. *J. Appl. Cryst.* **1986**, 105-107.

5 Supplementary data

5.1 ^{1}H and ^{13}C NMR spectra of 8

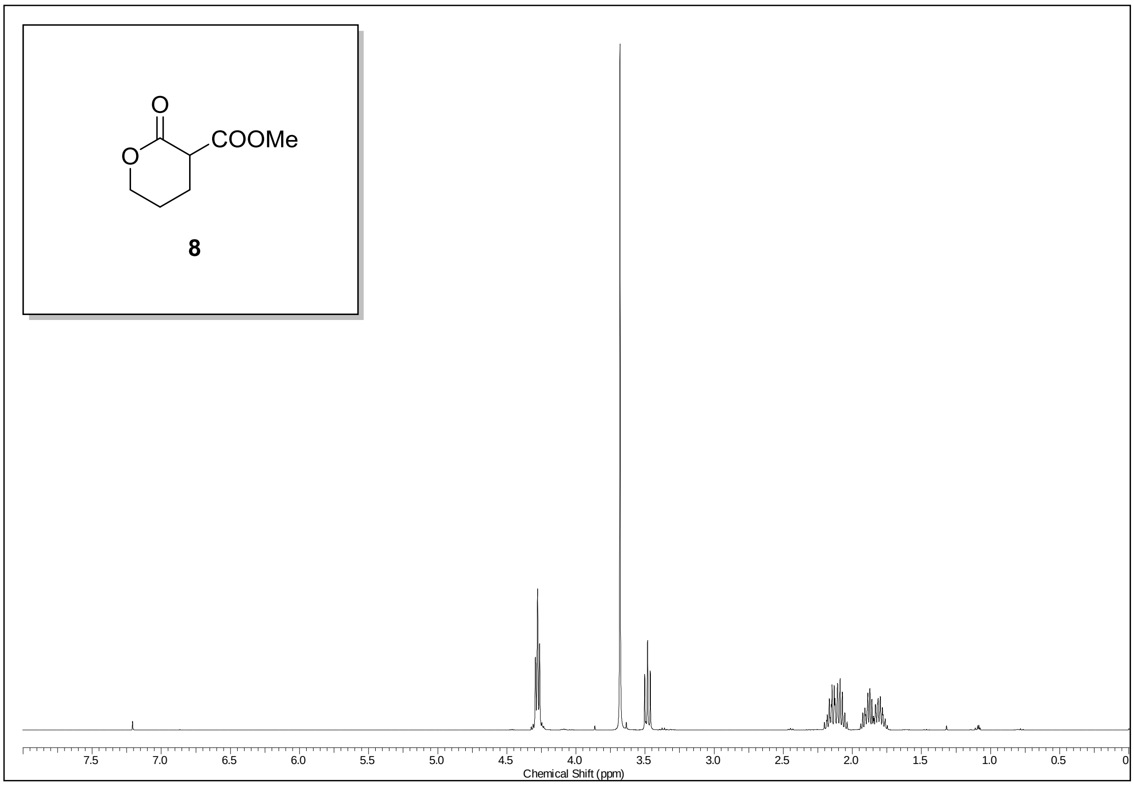

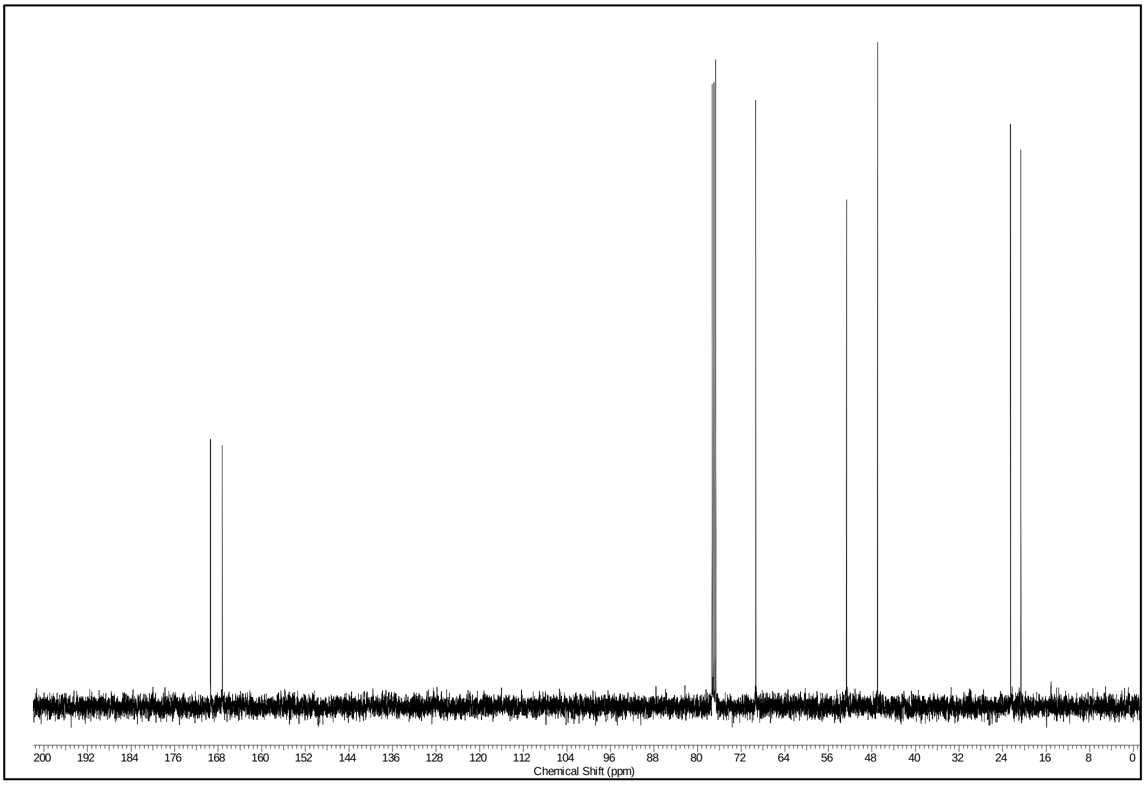

5.2 ^{1}H and ^{13}C NMR spectra of 13

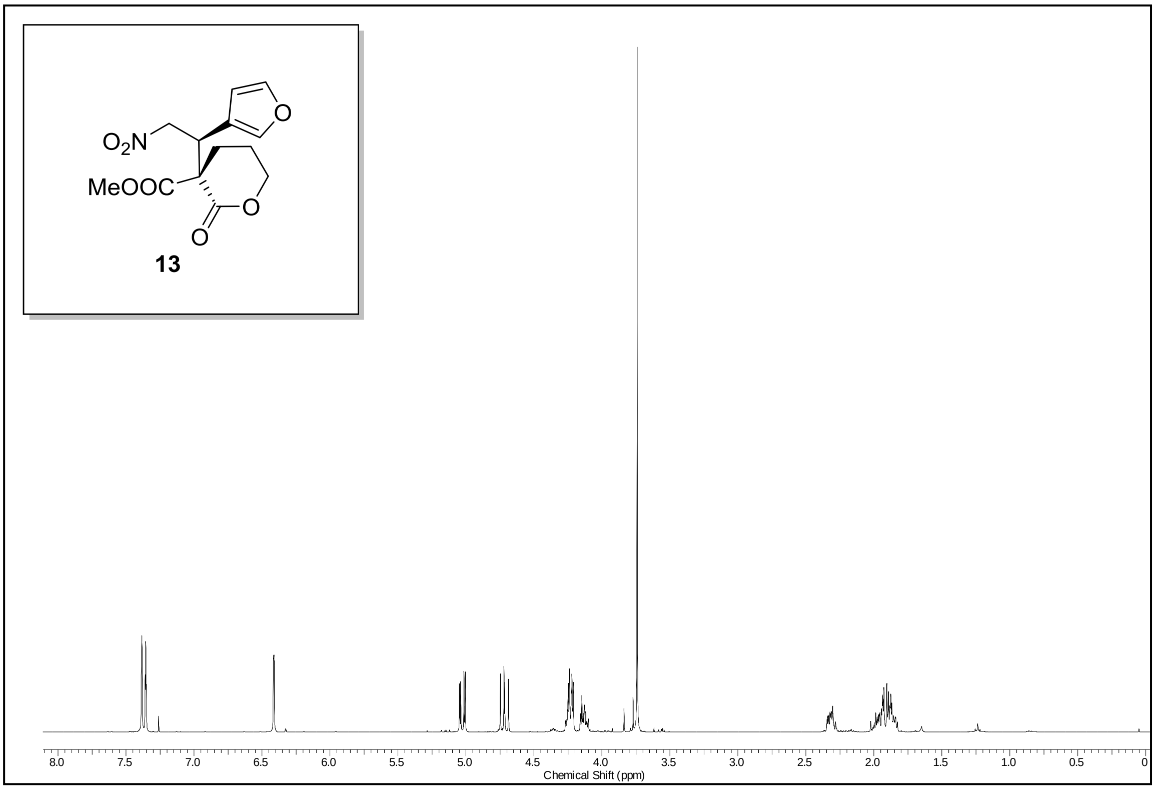

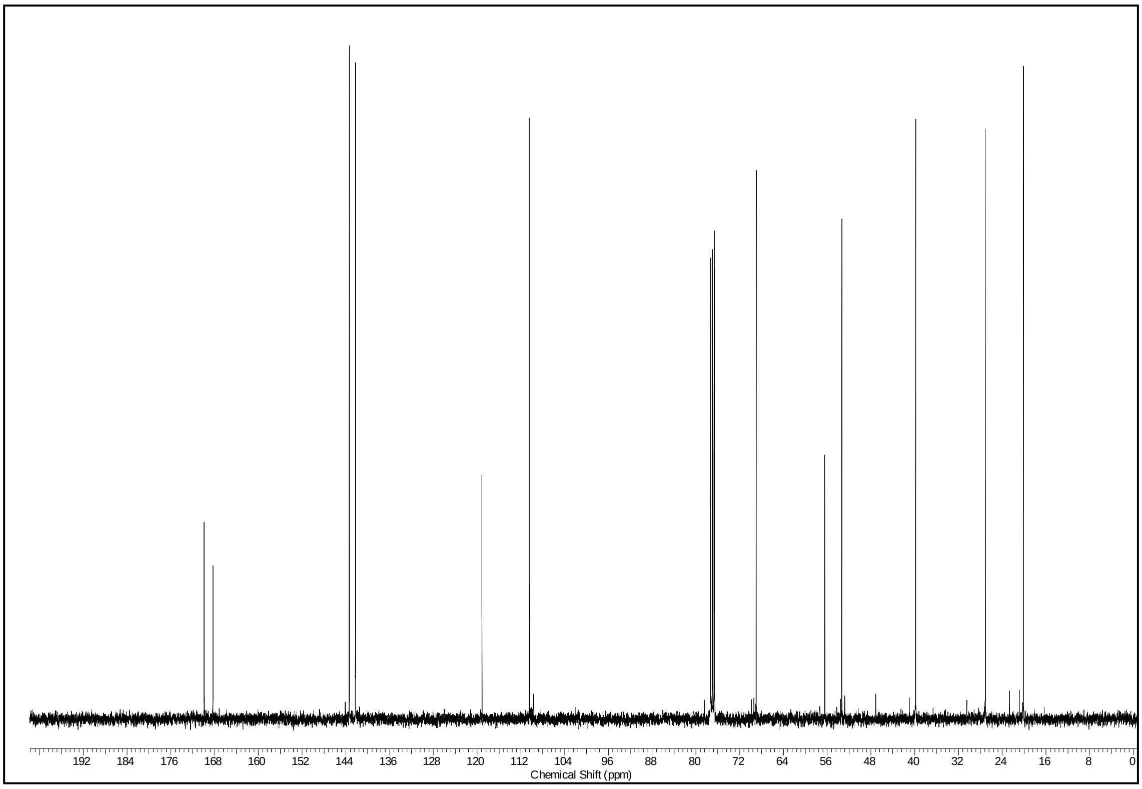

5.3 ^{1}H and ^{13}C NMR spectra of 14

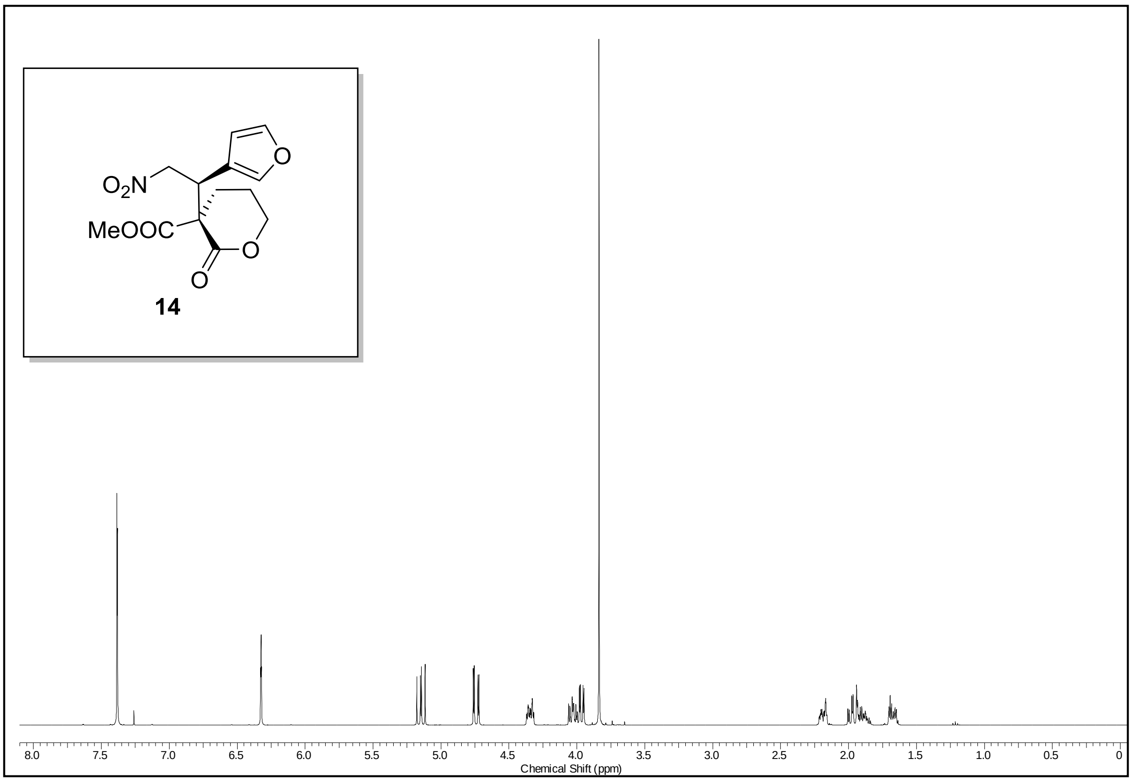

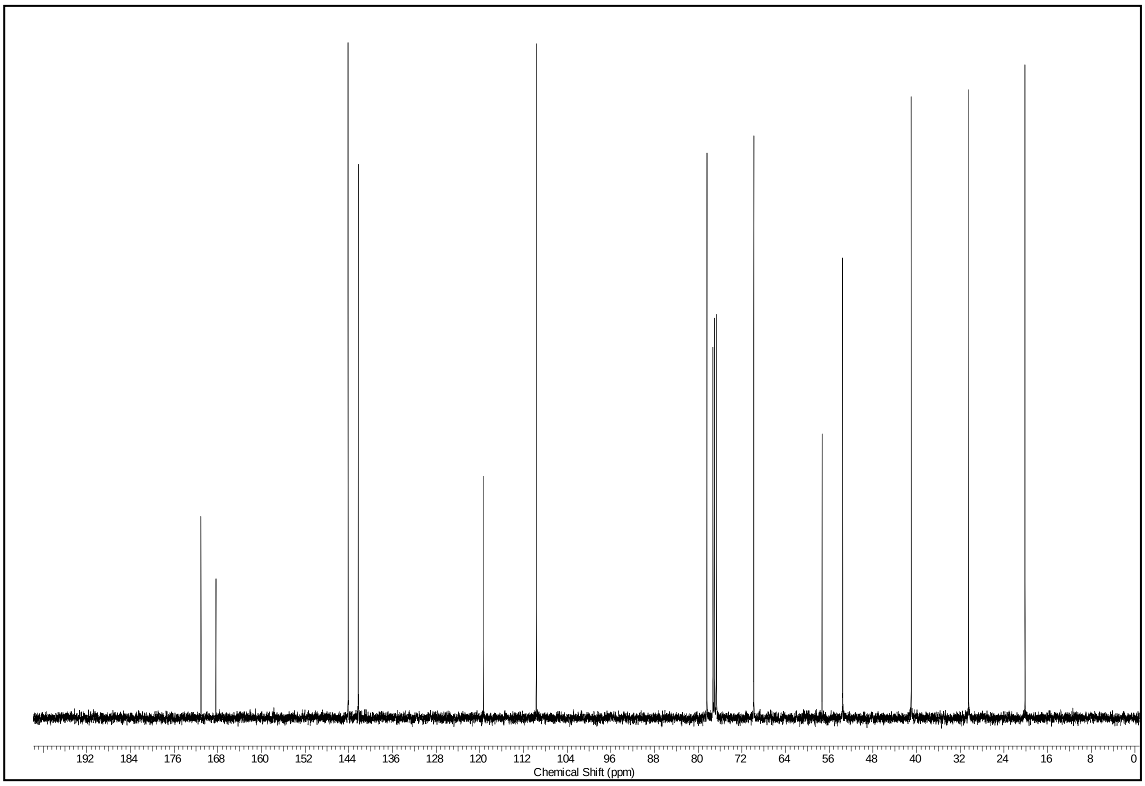

5.4 ^{1}H and ^{13}C NMR spectra of 15

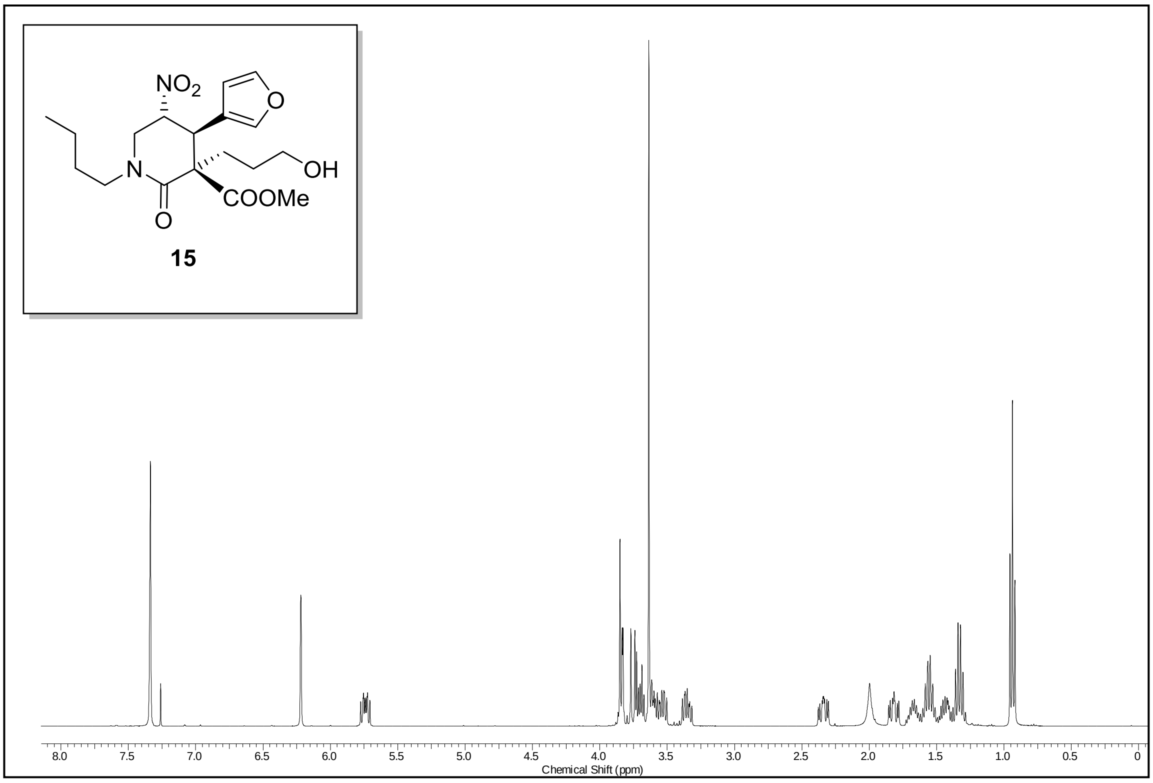

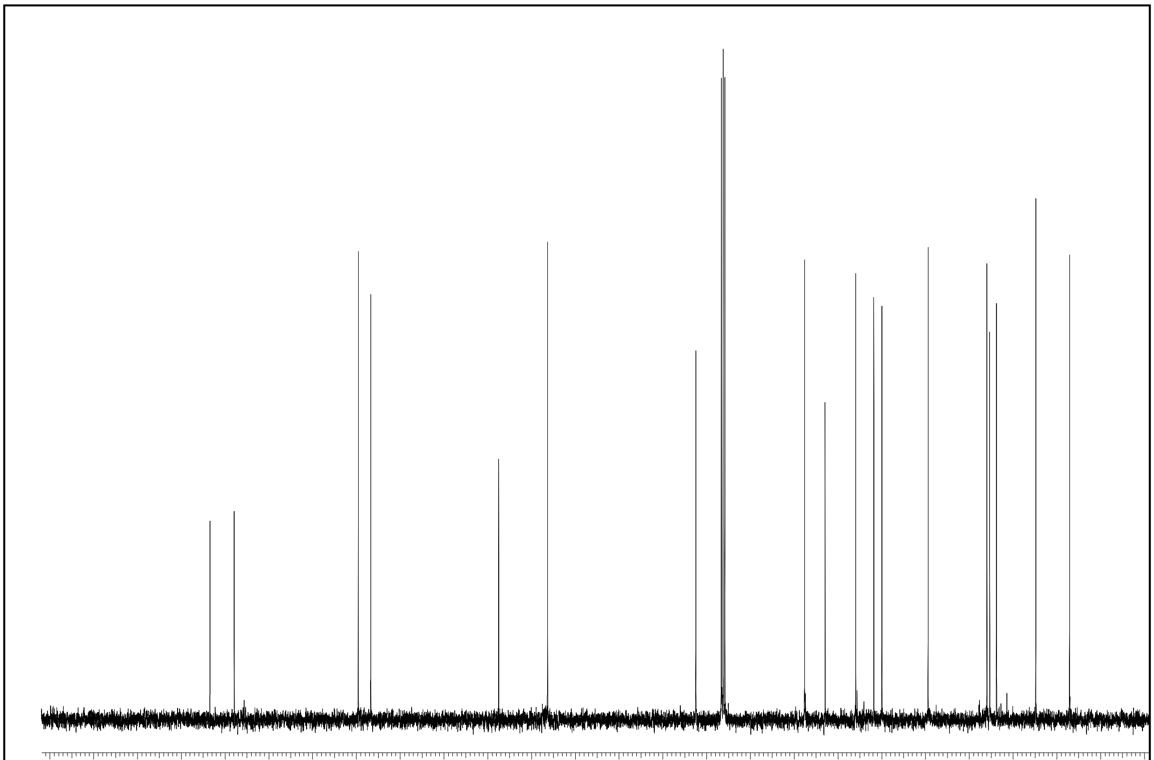

5.5 ^{1}H and ^{13}C NMR spectra of 7

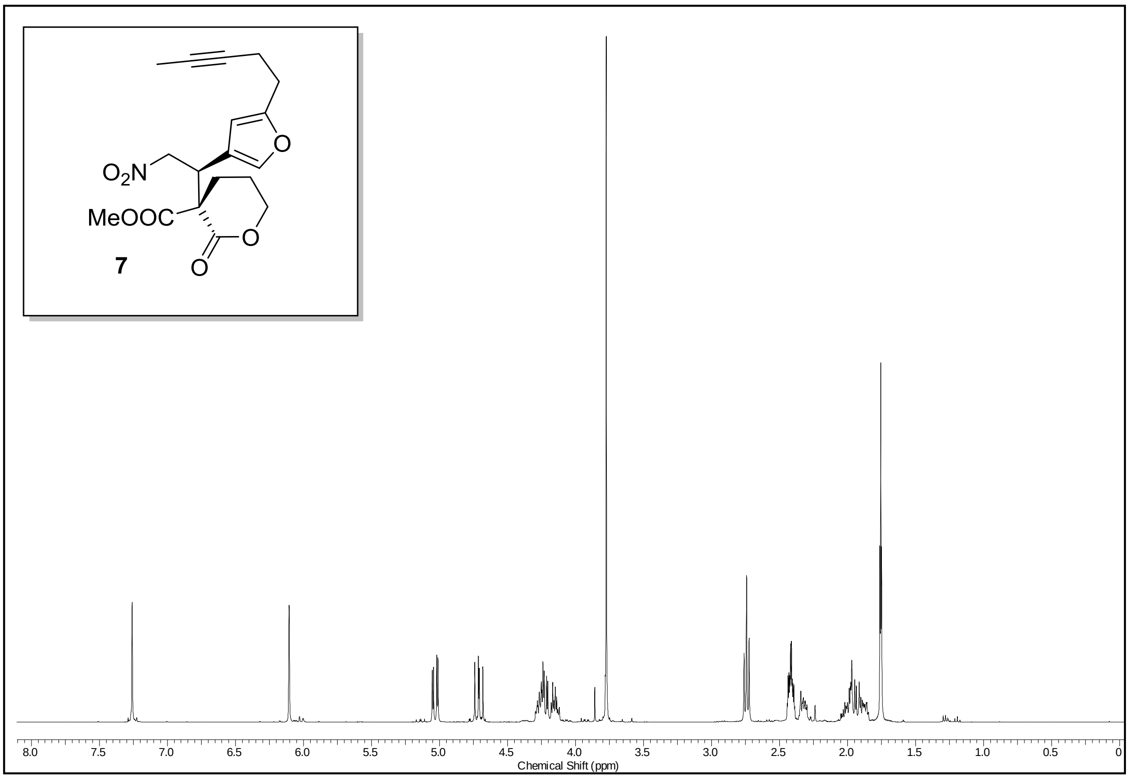

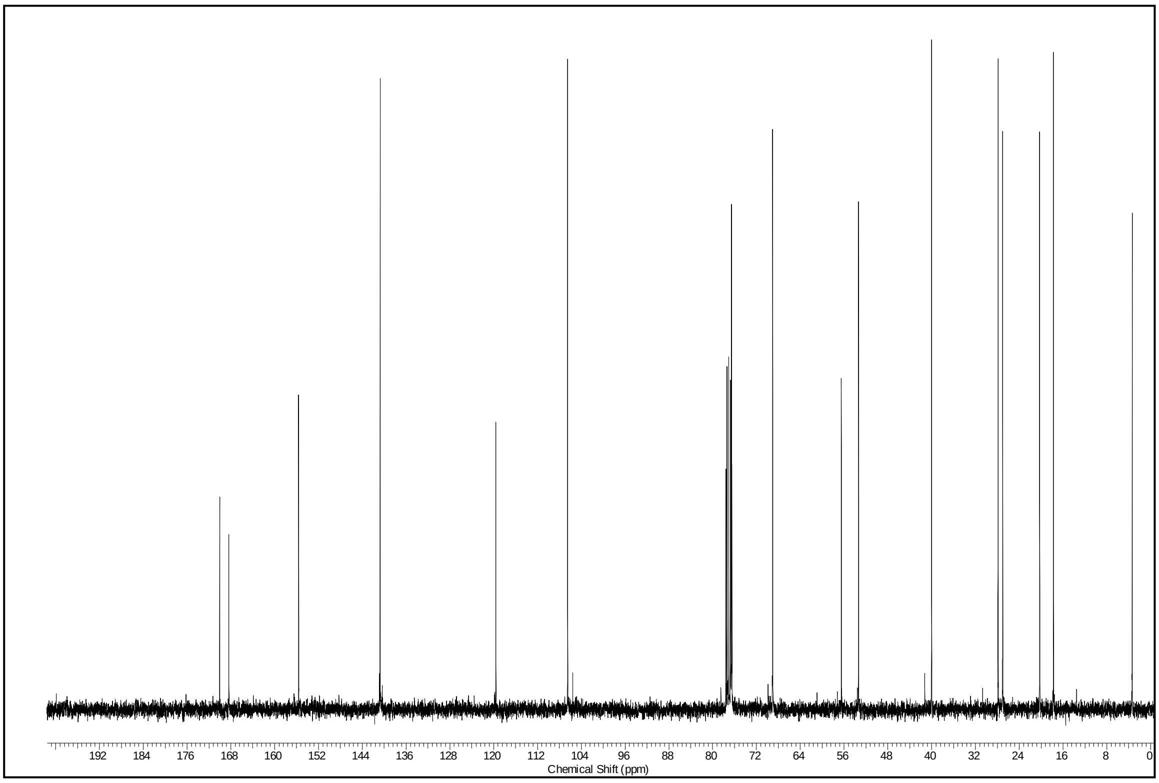

5.6 ¹H and ¹³C NMR spectra of 6

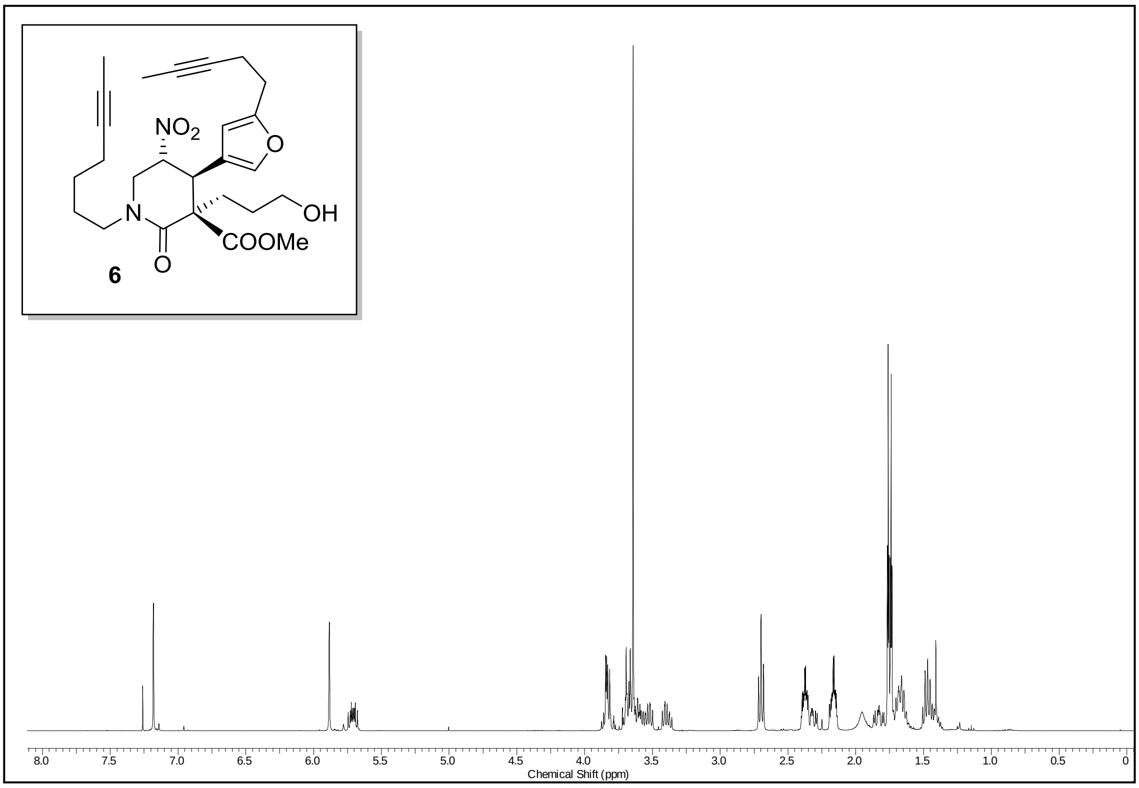

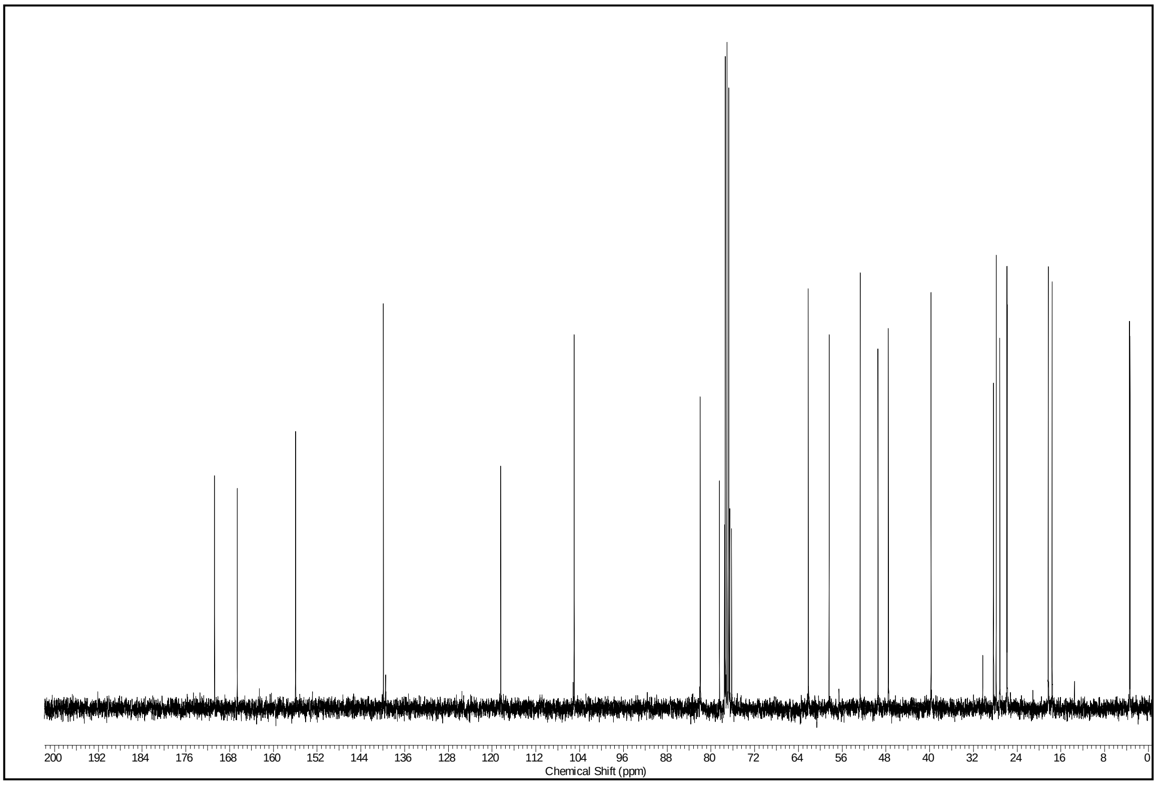

5.7 ¹H and ¹³C NMR spectra of 17

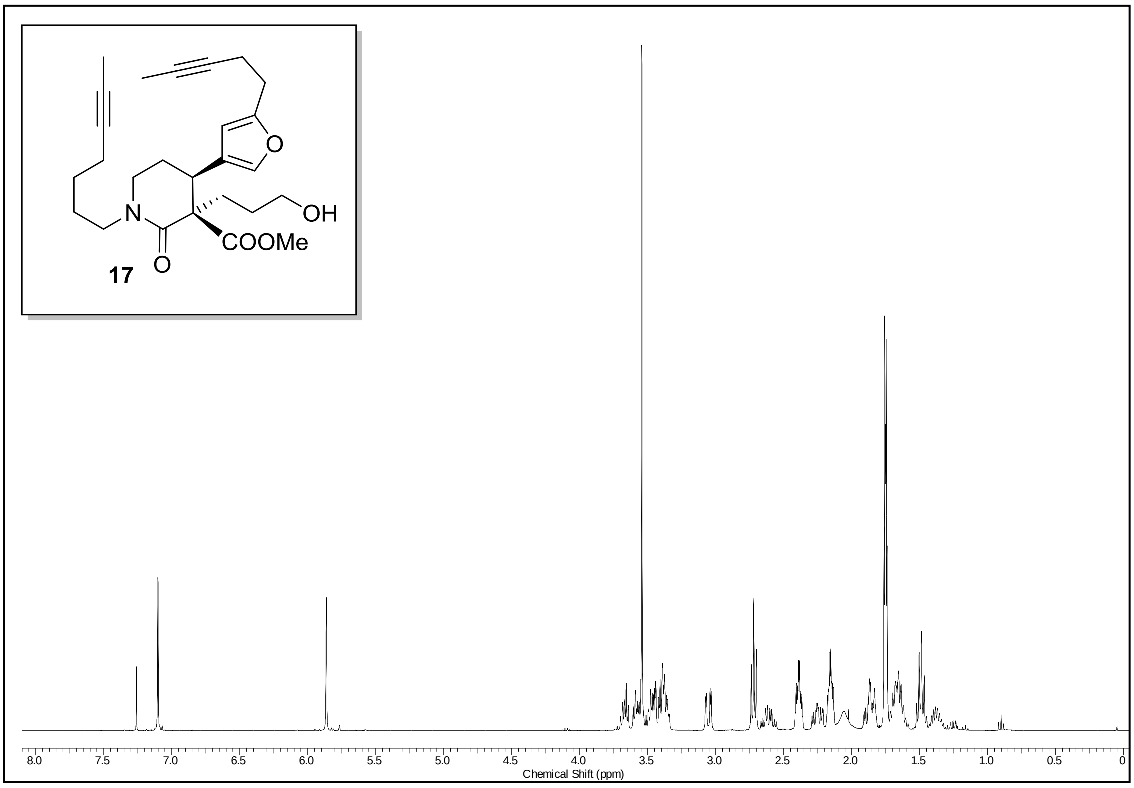

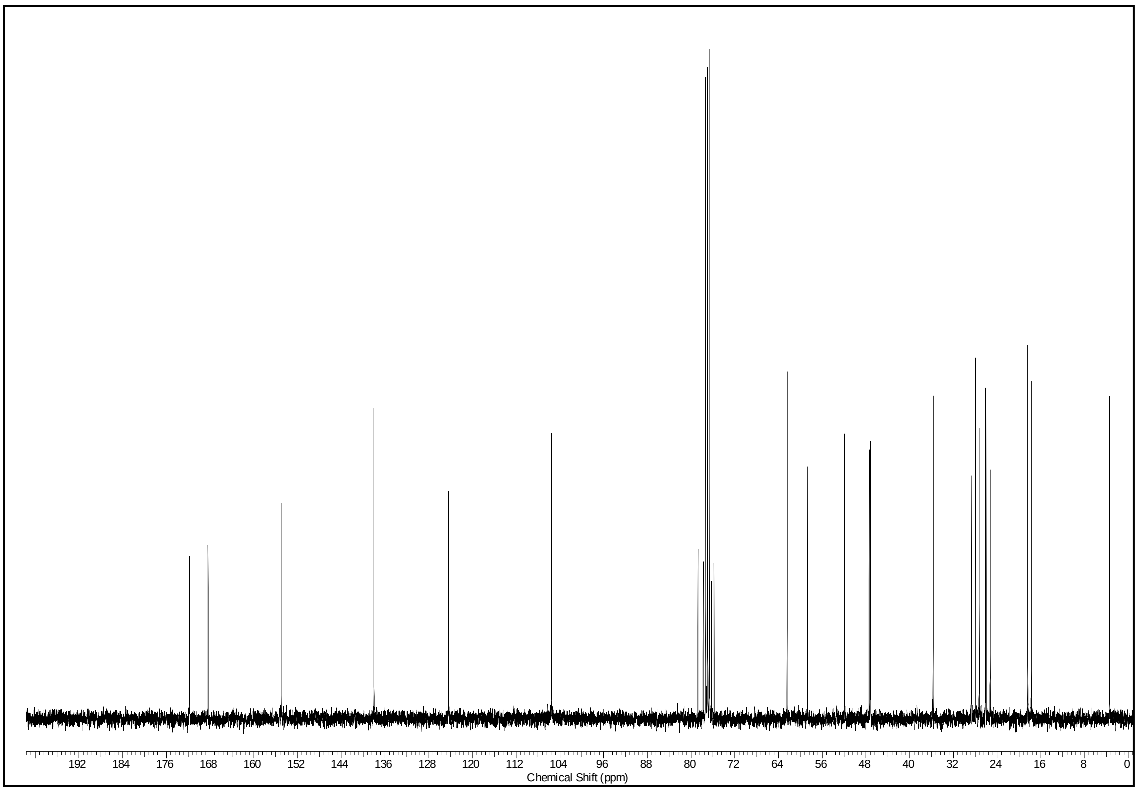

5.8 ¹H and ¹³C NMR spectra of 18

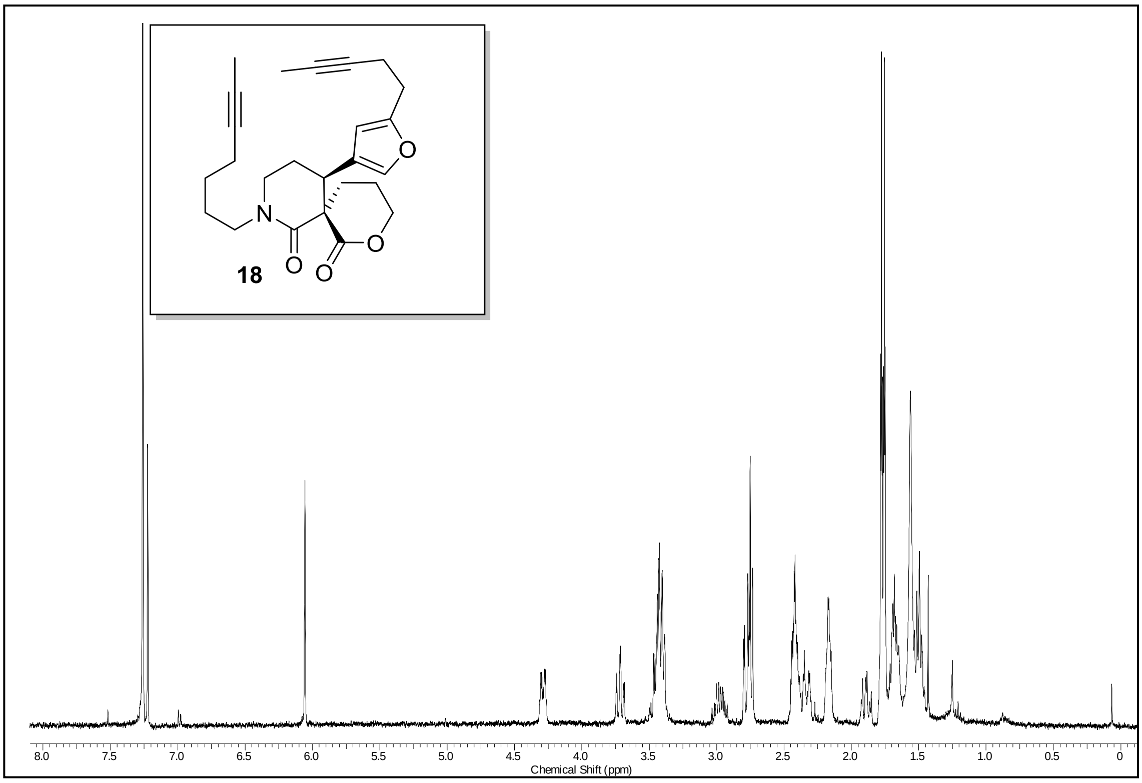

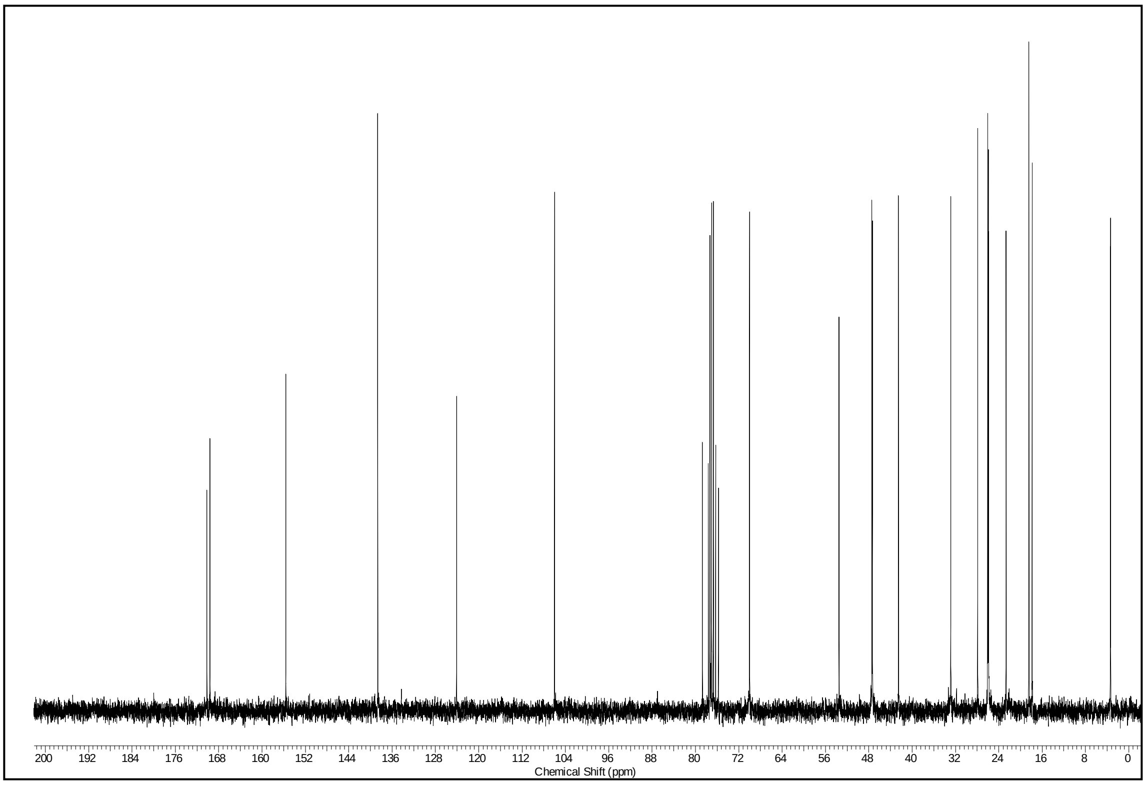

5.9 ^{1}H and ^{13}C NMR spectra of 20

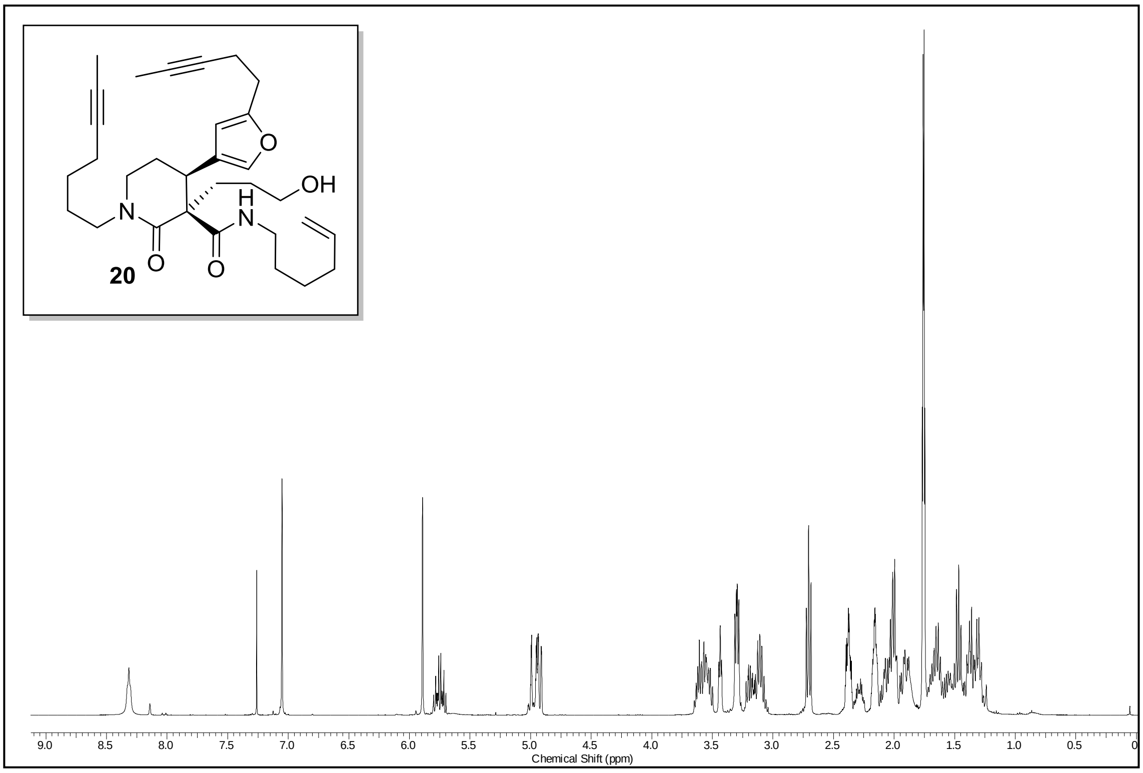

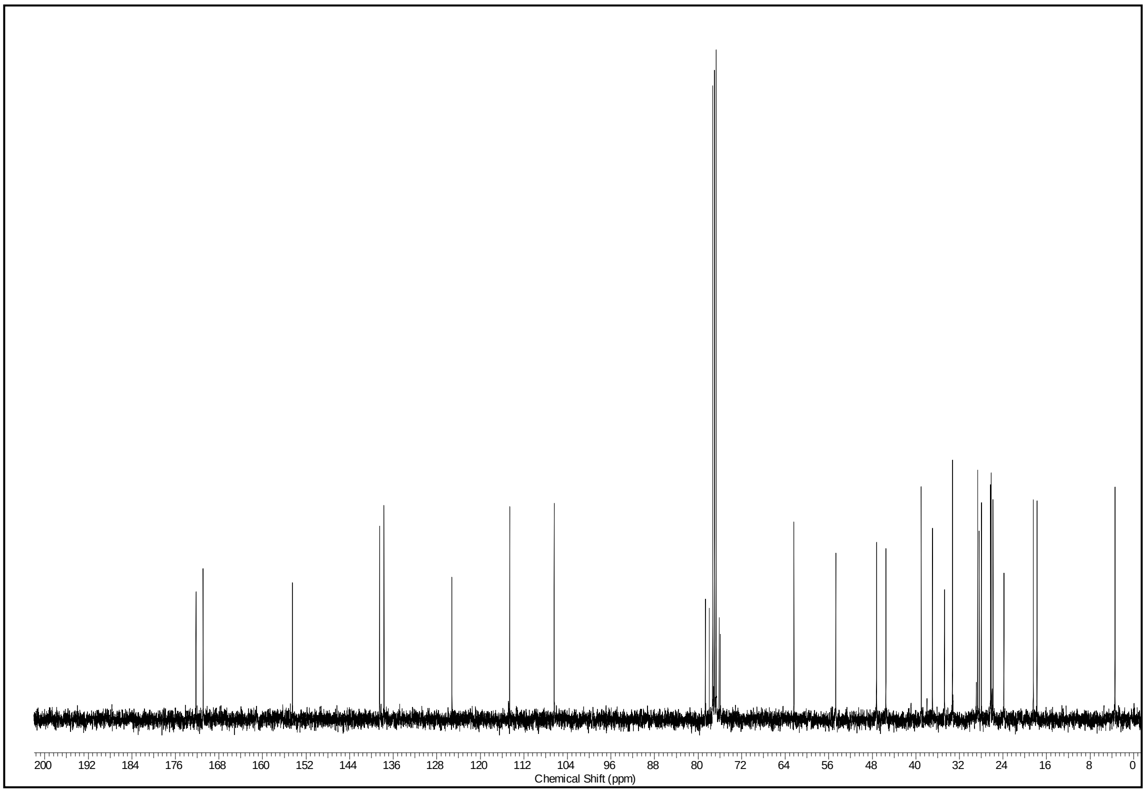

5.10 ^{1}H and ^{13}C NMR spectra of 21

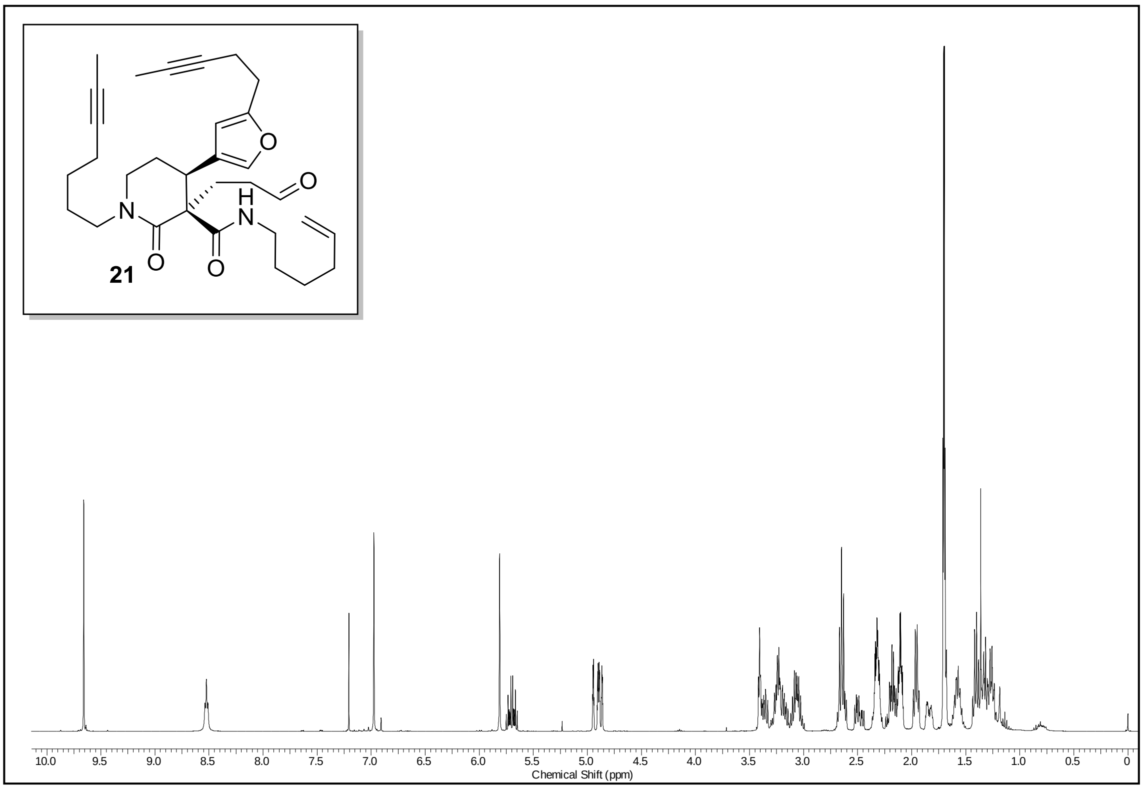

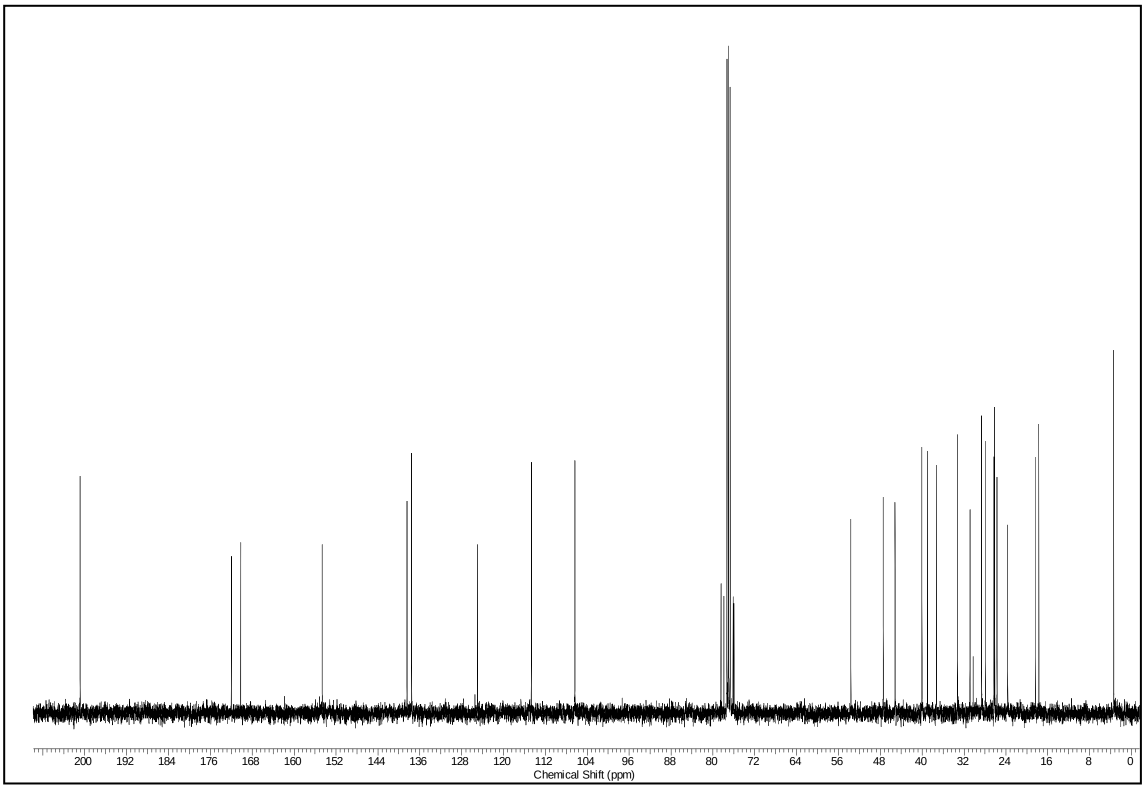

5.11 ¹H and ¹³C NMR spectra of 5

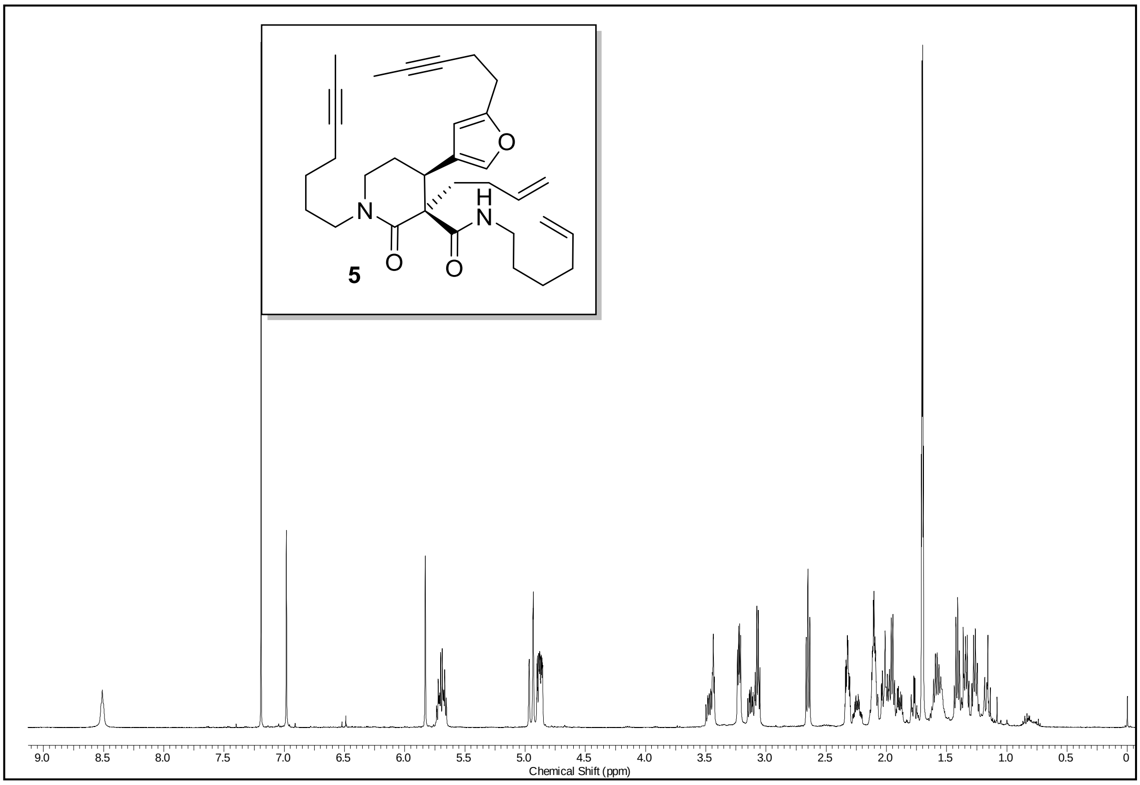

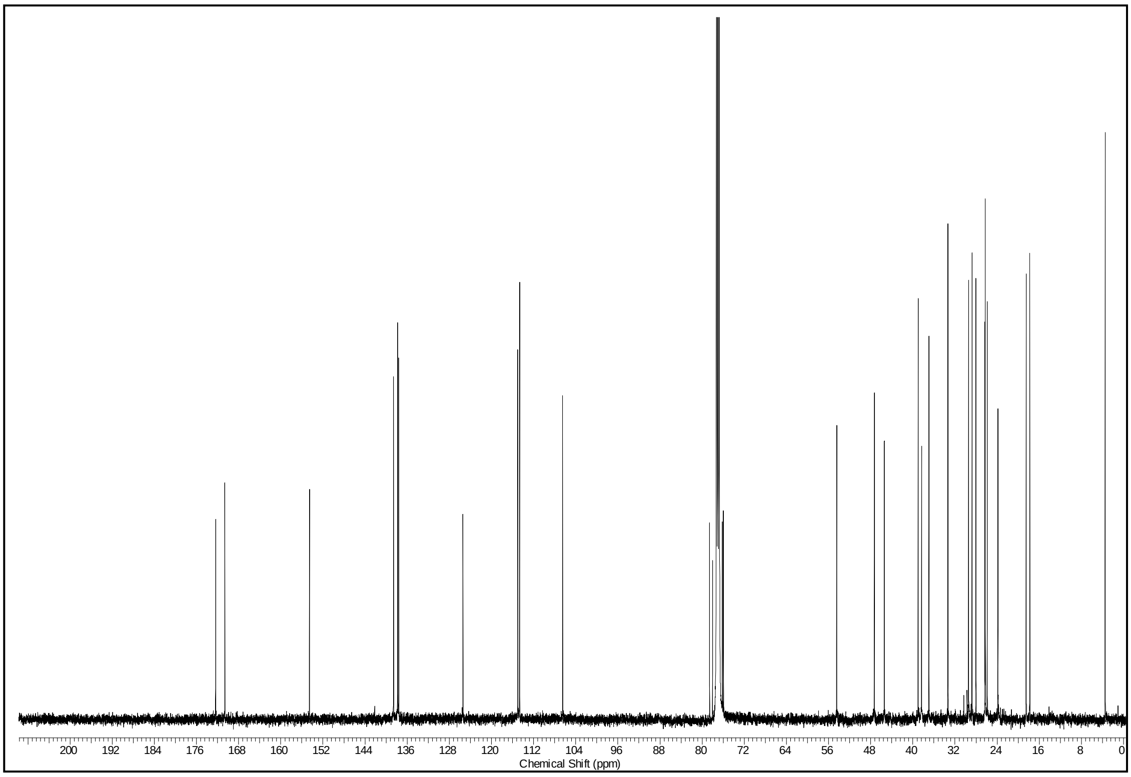

5.12 HPLC chromatogram of 13

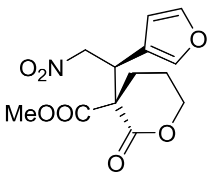

13

(Chiralpak AS-H, hexane/isopropanol = 80/20, 1 mL/min)

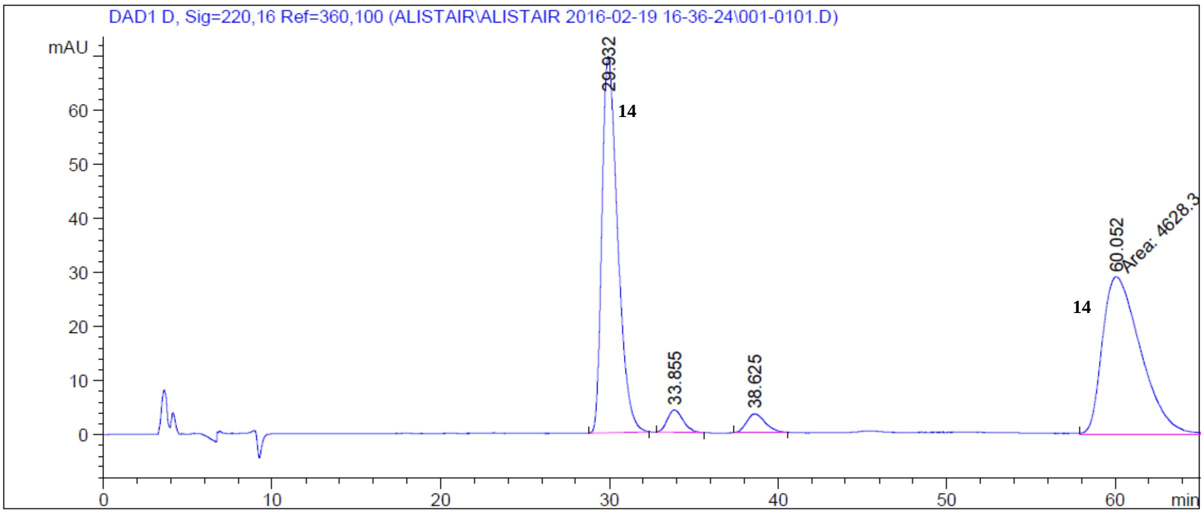

Signal 4: DAD1 D, Sig=220,16 Ref=360,100

Peak #	RetTime [min]	Type	Width [min]	Area [mAU*s]	Height [mAU]	Area %
1	29.932	BB	1.0007	4521.55371	69.70821	46.6530
2	33.855	BB	0.7836	269.92154	4.14546	2.7850
3	38.625	BB	0.9368	272.09949	3.47006	2.8075
4	60.052	MM	2.6360	4628.30469	29.26389	47.7545

Totals : 9691.87943 106.58762

Enantiomerically enriched (90:10 er)

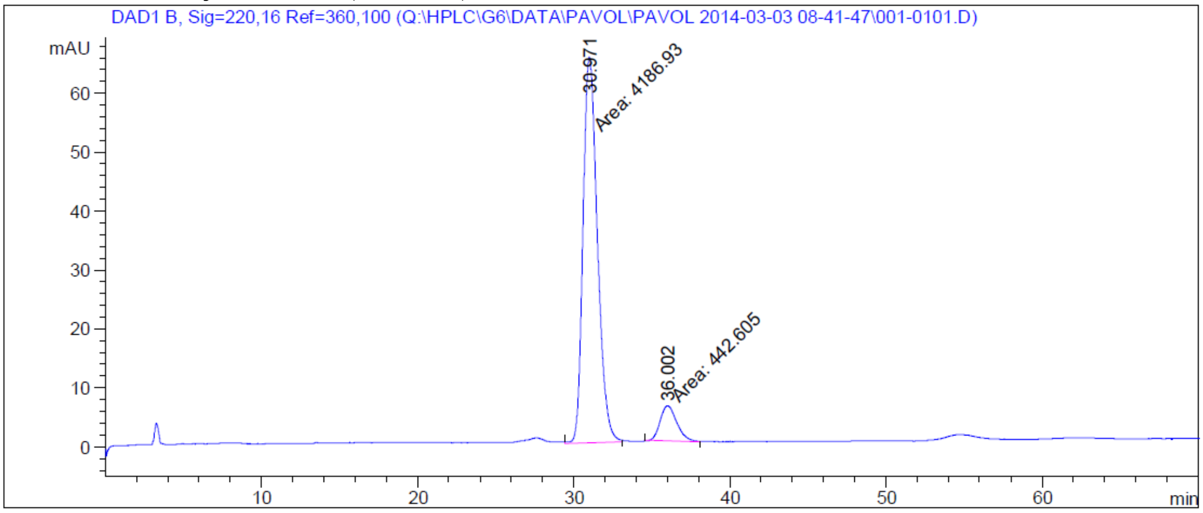

Signal 2: DAD1 B, Sig=220,16 Ref=360,100

Peak #	RetTime [min]	Type	Width [min]	Area [mAU*s]	Height [mAU]	Area %
1	30.971	MM	1.0656	4186.92627	65.48839	90.4395
2	36.002	MM	1.2354	442.60526	5.97118	9.5605

Totals : 4629.53152 71.45957

5.13 HPLC chromatogram of 6

(Chiralpak OD-H, hexane/isopropanol = 50/50, 0.6 mL/min)
Racemic

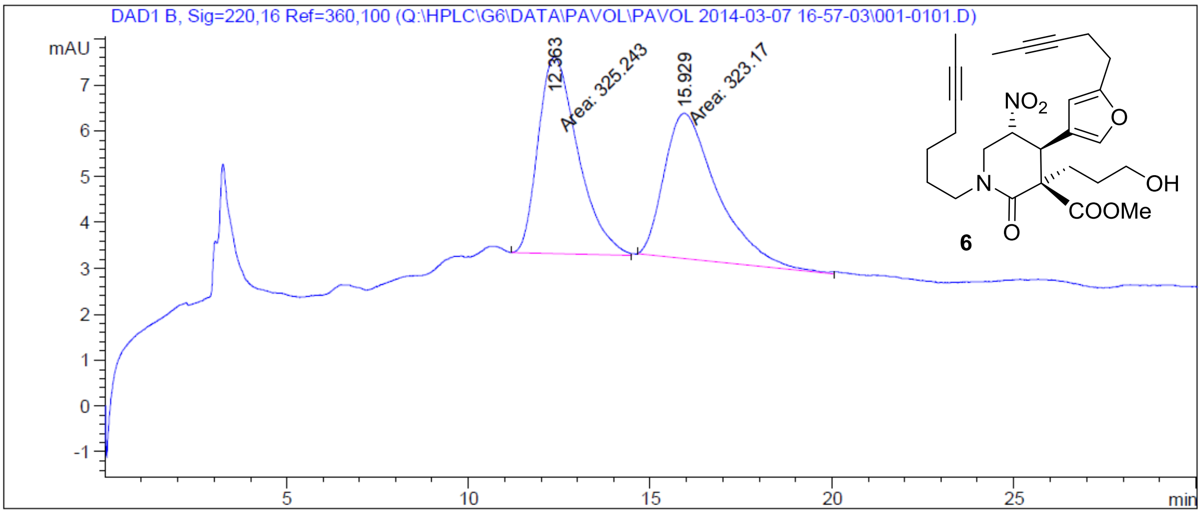

Signal 2: DAD1 B, Sig=220,16 Ref=360,100

Peak #	RetTime [min]	Type	Width [min]	Area [mAU*s]	Height [mAU]	Area %
1	12.363	MM	1.2655	325.24335	4.28362	50.1599
2	15.929	MM	1.7001	323.16968	3.16806	49.8401
Totals :				648.41302	7.45168	

Enantiomerically enriched (91:9)

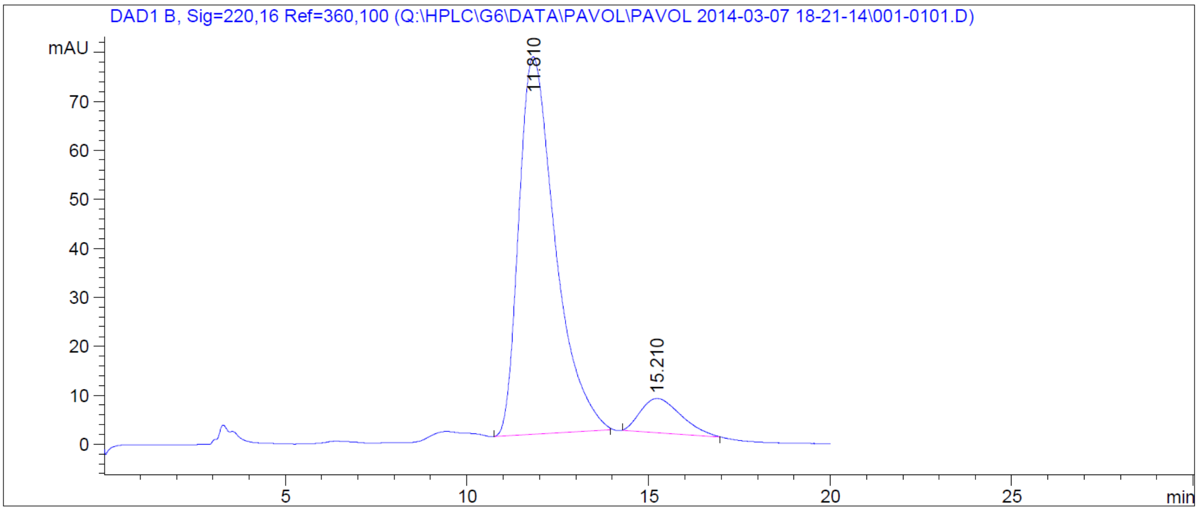

Signal 2: DAD1 B, Sig=220,16 Ref=360,100

Peak #	RetTime [min]	Type	Width [min]	Area [mAU*s]	Height [mAU]	Area %
1	11.810	BB	1.0596	5389.31348	77.08876	90.8487
2	15.210	BB	0.9314	542.87494	6.96469	9.1513
Totals :				5932.18842	84.05345	
